# Semitransparent Perovskite Solar Cells with Ultrathin Protective Buffer Layers

**DOI:** 10.1021/acsaem.3c00735

**Published:** 2023-10-05

**Authors:** Erica Magliano, Paolo Mariani, Antonio Agresti, Sara Pescetelli, Fabio Matteocci, Babak Taheri, Antonio Cricenti, Marco Luce, Aldo Di Carlo

**Affiliations:** † C.H.O.S.E. (Center for Hybrid and Organic Solar Energy), Electronic Engineering Department, University of Rome Tor Vergata, Via del Politecnico 1, 00133, Rome, Italy; ‡ ENEA - Centro Ricerche Frascati, Via Enrico Fermi, 45, 00044, Frascati, Rome, Italy; § Istituto di Struttura della Materia (CNR-ISM) National Research Council, via del Fosso del Cavaliere 100, 00133, Rome, Italy

**Keywords:** semitransparent perovskite solar cells, sputtering damage, S-shape, buffer layers, vanadium oxide, molybdenum oxide

## Abstract

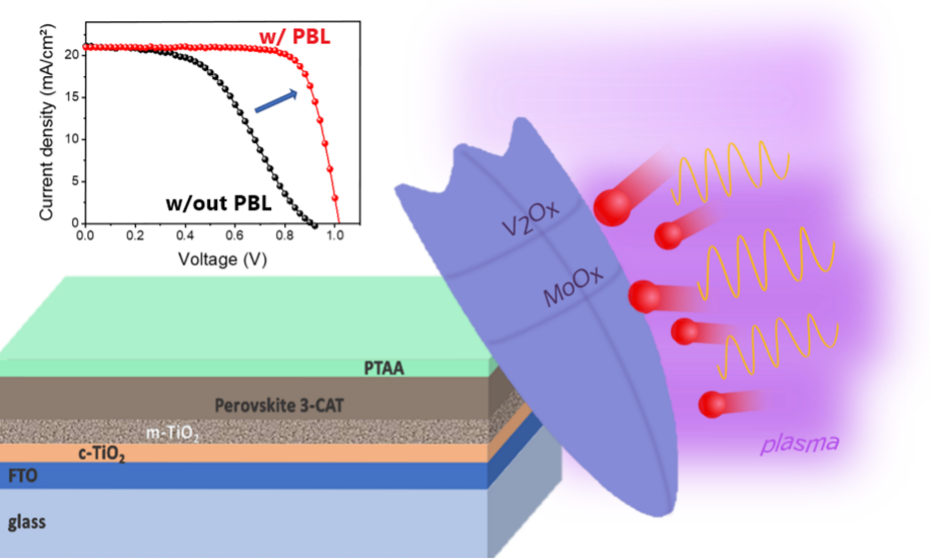

Semitransparent perovskite solar cells (ST-PSCs) are increasingly important in a range of applications, including top cells in tandem devices and see-through photovoltaics. Transparent conductive oxides (TCOs) are commonly used as transparent electrodes, with sputtering being the preferred deposition method. However, this process can damage exposed layers, affecting the electrical performance of the devices. In this study, an indium tin oxide (ITO) deposition process that effectively suppresses sputtering damage was developed using a transition metal oxides (TMOs)-based buffer layer. An ultrathin (<10 nm) layer of evaporated vanadium oxide or molybdenum oxide was found to be effective in protecting against sputtering damage in ST-PSCs for tandem applications, as well as in thin perovskite-based devices for building-integrated photovoltaics. The identification of minimal parasitic absorption, the high work function and the analysis of oxygen vacancies denoted that the TMO layers are suitable for use in ST-PSCs. The highest fill factor (FF) achieved was 76%, and the efficiency (16.4%) was reduced by less than 10% when compared with the efficiency of gold-based PSCs. Moreover, up-scaling to 1 cm^2^-large area ST-PSCs with the buffer layer was successfully demonstrated with an FF of ∼70% and an efficiency of 15.7%. Comparing the two TMOs, the ST-PSC with an ultrathin V_2_O_*x*_ layer was slightly less efficient than that with MoO_*x*_, but its superior transmittance in the near infrared and greater light-soaking stability (a *T*_80_ of 600 h for V_2_O_*x*_ compared to a *T*_80_ of 12 h for MoO_*x*_) make V_2_O_*x*_ a promising buffer layer for preventing ITO sputtering damage in ST-PSCs.

## Introduction

Solar photovoltaic (PV) demand has been increasing over the years since it represents one of the foremost renewable energies in the decarbonization of the world’s energy system.^1^ Nowadays, silicon-based technology is leading the PV market, due to its superior cost-performance, stability and robustness when compared with other PV technologies.^2^ The back-contact heterojunction (BCHJ) crystalline Si (c-Si) solar cells, developed by LONGi Green Energy Technology Co., has achieved a record efficiency of 26.81%,^3^ which is approaching the theoretical limit (29.1%).^4,5^

Tandem solar cells (TSCs), which are made up of two light absorbers with different band gaps,^6,7^ represent the most straightforward option for boosting and overcoming the single-junction efficiency limit.^8^ Metal halide perovskite materials have been revealed to be a promising candidate for silicon in tandem configuration, and the theoretical limit can increase up to 40%.^9,10^ Perovskite materials show ideal properties for tandem applications, such as very sharp absorption edge, tunable bandgap, and potentially low-cost fabrication.^11−14^ These features allow the combination of perovskite solar cell (PSC) technology with low-bandgap semiconductors, such as Si.^15^ In this context, the semitransparent perovskite top cell plays a crucial role in the final tandem device efficiency, and its optimization in terms of electrical performance and optical transparency is pivotal.^16^

Beside tandem solar cell applications, semitransparent perovskite solar cells (ST-PSCs) attracted attention because of their potential application in see-through building-integrated photovoltaics (BIPVs).^17^ BIPVs represent a promising option to incur building energy demand. Based on the specific photovoltaic application, the required transparency level of ST-PSCs can vary: on one hand, for BIPVs, a high transmittance in the visible range (380–780 nm) is required; on the other hand, for tandem applications with low-bandgap materials, the range of interest is in the near-infrared region (NIR) of the solar spectrum (780–1200 nm).^18−20^ Nevertheless, opaque PSCs hold the highest power conversion efficiencies (PCEs) in perovskite-based technology as compared with ST-PSCs, mainly due to the reflectivity of the metallic counter electrode which increases the absorption in the perovskite film.^21^ In order to overcome this lack, ST-PSCs must be designed to increase light-trapping and manage light-absorption without impairing the transparency. Since transparent electrodes are used as contacts, a trade-off between transparency and conductivity must be found. Metallic films, such as Au, Al or Ag, can still be used as transparent electrodes, even though an ultrathin layer must be deposited and the conductivity is jeopardized. However, metallic films can limit the transparency and thus decrease the absorption in the cells. In this context, metal nanowires (NWs) show high transparency, even though their film uniformity and thermal/chemical stability must be improved.^16,22^ Carbon-based materials, such as graphene or carbon nanotubes (CNTs), can be followed as an alternative approach since they exhibit excellent electrical properties, light transmittance, and stability. However, scale-up of the process is currently the main limitation of carbon-based electrodes.^23^

Owing to their high electrical mobility combined with high transparency, transparent conductive oxides (TCOs) are the most common electrodes for ST-PSC and in several other applications.^22^ TCOs, such as indium tin oxide (ITO) or aluminum zinc oxide (AZO), are usually deposited by direct current (DC) or radio frequency (RF) magnetron sputtering. Recently, pulsed laser deposition (PLD) is receiving more attention in TCOs fabrication because of its less-damaging peculiar property in ST-PSCs.^24^ However, the high throughput of this technique has yet to be demonstrated. Sputtering is a physical vapor deposition (PVD) technique and takes all the advantages of this class of fabrication processes, such as high purity, uniformity, and thickness-control of the deposited film. However, low-medium vacuum range (in the order of 10^–3^ mbar) is necessary to carry out the process, which might induce higher impurities incorporation into the deposited film. During the process, in addition to the sputtered target particles, other species can strike the substrate, such as negative ions (up to 400 eV) and high energy electrons (up to 200 eV). This phenomenon, together with plasma-luminescence and induced heat, can hardly affect sensitive layers and surface or bulk damage of the substrate can occur.^22,25,26^ In ST-PSCs, the sputtering damage can cause an increased series resistance and an increment of the energy barrier height at the interface between the electron or hole transport layer (ETL or HTL) and the sputtered TCO. This can hinder the carrier transport resulting in the typical “*S*-shape” affecting the device current–voltage (*I*–*V*) curve.^16,22,27,28^ To avoid the sputtering damage issue, process parameters can be tuned to optimize a soft sputtering process,^29−31^ or a protective buffer layer (PBL) can be introduced.^32−34^

In this work, we evaluated the effects of tuning the power density and the deposition time during ITO sputtering as well as the effect of introducing a PBL to mitigate the sputtering damage. The second approach is shown to successfully suppress damage to the underlying layers. We investigated the effectiveness of sputtering damage prevention of two different evaporated transition metal oxides (TMOs), molybdenum oxide (MoO_*x*_) and vanadium oxide (V_2_O_*x*_). MoO_*x*_ has been extensively used in n-i-p ST-PSCs as buffer layer.^35−38^ On the other hand, V_2_O_*x*_ buffer layer has been deposited only via atomic layer deposition (ALD) so far, leading to efficiencies up to 13.4% in Raiford et al.’s work.^39^ It has also been employed as PBL in perovskite/silicon tandem in the study of Aydin and co-workers.^40^ In these studies, thicknesses between 9 and 10 nm were employed. We point out that ALD is commonly used for the buffer layer deposition in ST-PSCs. ALD has the advantages of atomic plane resolution and step coverage, even though the high energy and material consumption compared to thermal evaporation can limit its applicability.^41−44^ During an ALD process, nearly 60% of the precursors’ amount is wasted, resulting in a poor material efficiency utilization.^45^ Moreover, its commercial use is limited by the high cost of the precursors. Additionally, the carrier gases can damage the underlying layers,^41,46,47^ and incorporation of water or oxygen can occur as well.^48^ ALD is based on chemical reactions of the precursor gases onto the substrate, which determine the deposition time. For this reason, ALD is characterized by the slowest deposition rate among the other deposition techniques, as reported in (45). This aspect can critically hinder ALD’s industrial applicability since it could slow down the production line or increase the capital expenditure (CAPEX). Moreover, in an industrial cluster for PSCs fabrication, thermal sublimation could be employed to deposit the other layers of the device stack (such as the HTL, ETL, as well as the perovskite layer) in a multisource evaporation chamber without breaking vacuum for fully vacuum-processed PSCs. Hence, thermal sublimation represents a promising technique for buffer layer deposition that is not hampering the path of PSCs toward upscaling and industrialization.

In this paper, we focus on the simple thermal evaporation of PBL showing that an ultrathin layer of MoO_*x*_ is sufficient to hinder the sputtering damage issue. This investigation has been extended to vanadium oxide as well,^40,49^ and to the best of the authors’ knowledge, this is the first time that evaporated vanadium oxide is employed in PSCs as buffer layer. V_2_O_*x*_ shows buffer characteristics similar to those of MoO_*x*_ but with higher stability properties: ST-PSCs with sputtered ITO and a V_2_O_*x*_ buffer layer retain 80% of its initial efficiency under continuous light-soaking stress test for 600 h, while MoO_*x*_-based devices retain efficiency for only 12 h.

Differently to previous works on TMOs where the impact of the sputtering process increases the optical losses of the oxides,^50^ we observed a negligible parasitic absorption. An investigation on the oxidation state, on the oxygen-deficiency effect and on crystalline morphology is also reported and is then related to the power conversion efficiency of the devices. We examined the influence of PBLs’ thickness in terms of electrical performance in the ST-PSCs. For both tested TMOs, an ultrathin layer (<10 nm) was found to be effective in mitigating the sputtering damage effects. This successful strategy allowed us to obtain a remarkable reduction of the series resistance and an excellent improvement of the fill factor (FF), up to 76.2%. The PBL was demonstrated to effectively mitigate the sputtering damage in thinner-perovskite based ST-PSCs for BIPV, as well as in large area (active area ≈1 cm^2^) ST-PSCs. This result paves the way for up-scaling and for tandem and see-through PV application.

## Results and Discussion

In this study, we consider ST-PSCs based on a mesoscopic architecture with the following reference structure: glass/FTO/c-TiO_2_/m-TiO_2_/perovskite/PTAA/ITO. This architecture was chosen since it is among the most performing PSCs^51,52^ and the fabrication processes are well-known and consolidated.^53^

**Figure 1:**
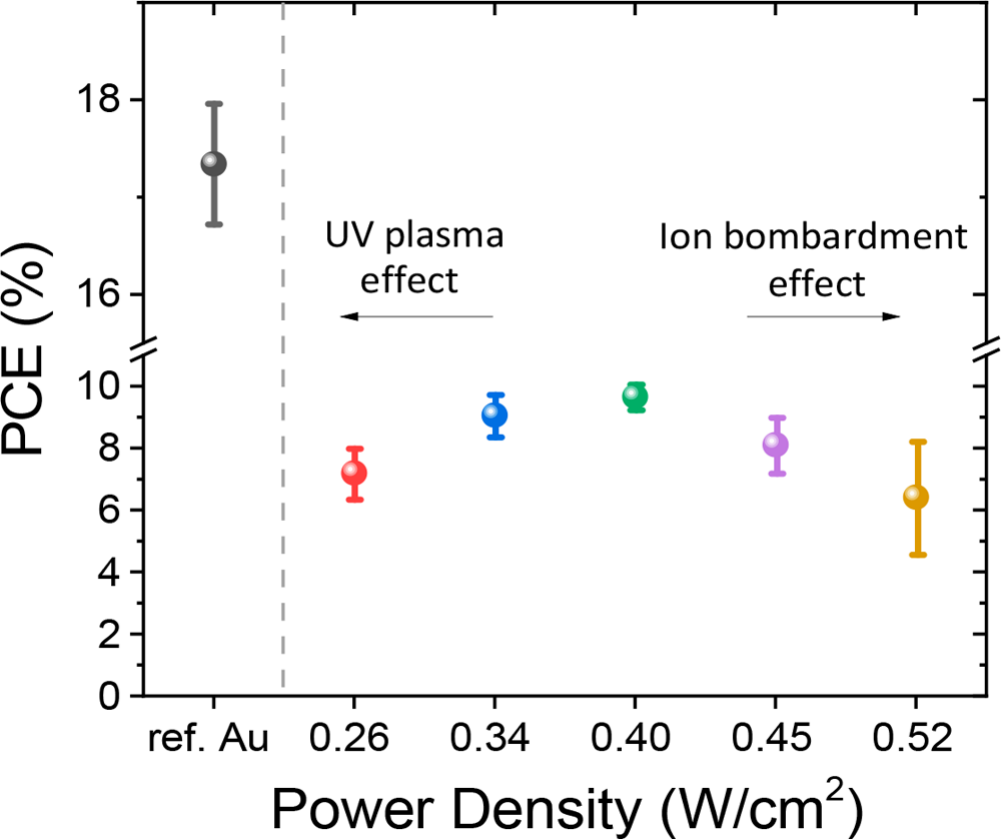
PCE as a function of the input power density of the sputtering system.

RF-sputtered ITO was employed as the top electrode for the ST-PSCs. All of the process parameters can be found in the Device Fabrication section. One of the main parameters that features the sputtering process is the applied RF power density, which is defined as the ratio between the RF power and the target area. It has been reported that it holds a crucial role in the determination of the optical and electrical properties of the ITO film and its crystallinity.^54−56^ In this work, the sputtering input power density was tuned to analyze the optoelectrical characteristics of the ITO film and the sputtering damage effect on the device performance. Different values were set: 0.26, 0.34, 0.39, 0.45, 0.52 W/cm^2^. The deposition time is controlled through the setting of the number of sputtering cycles, where one cycle represents a complete raster of the substrate under the rectangular ITO cathode. The cycle number was adjusted accordingly, in order to deposit always a 100 nm-thick ITO layer. Therefore, a similar sheet resistance is obtained for all the deposited ITO films, as reported in Table S1. No significant difference is shown in the transmittance spectra (Figure S1). The electrical performance of the devices was analyzed as a function of the input power density (Figure 1). The gold-based PSCs are also reported for comparison, and the trend of all the electrical parameters as a function of the RF power density can be found in Figure S2. The ITO was sputtered directly atop the PTAA layer acting as HTL in the investigated mesoporous n-i-p PSCs. The FF was the most affected parameter when tuning power density and number of cycles. The best FF values were observed at 0.34 and 0.40 W/cm^2^. While the open circuit voltage (*V*_OC_) was also affected reaching the highest values at 0.40 W/cm^2^, the short circuit current (*J*_SC_) was independent from the power density, showing a constant trend. This can be explained by the fact that the perovskite absorber was not varied. The power conversion efficiency (PCE) reached a maximum value when the input power density is 0.40 W/cm^2^. A low power ITO deposition might induce a less intense ion bombardment of the samples.^29,57^ However, since the ITO deposition rate decreases, the number of cycles is higher and the substrates are exposed for a longer period of time under the UV plasma, which is a sputtering damage source as well. The effect was evident in the FF of the devices processed at 0.26 W/cm^2^. Moreover, a reduction of *V*_OC_ was observed when the power density was reduced below 0.40 W/cm^2^. The reason might be found in a reduction of the ITO work function when exposed to UV plasma,^58^ which induces an increase of the barrier height. For higher power densities (higher than 0.40 W/cm^2^), a PCE decrease is also observed. In this case, the ion bombardment effect plays a crucial role by penalizing the device performance. Therefore, a process with a power density of 0.40 W/cm^2^ and 200 cycles was chosen.

The previous analysis was performed with a fixed Ar gas flow rate at 40 sccm during the process. It has been reported that a relatively small amount of oxygen gas might induce transmission and crystallinity improvements in the ITO deposited film.^59,60^ However, the oxygen vacancies’ concentration can diminish with the introduction of oxygen gas flow, which involves a decrease of ITO conductivity.^61−63^ Thus, optical and electrical ITO properties can be optimized with a proper oxygen concentration during the sputtering process. In this work, Ar and O_2_ gas flow rates in the sputter gas were tuned and the O_2_ gas percentage was varied from 0.3% to 4.8%. However, for all the tested concentrations, the sheet resistance values increased in comparison with the control process (Ar/O_2_ = 40 sccm/0 sccm) and lay in the kΩ/□ or MΩ/□ range as reported in Table S2. This can be explained by a decrease of the oxygen vacancies, which, in turn, causes a decrease of the ITO conductivity. However, when increasing the Ar gas flow rate up to 80 and 120 sccm and maintaining the O_2_ flux at 0.4 sccm, the sheet resistance values are not decreasing. The reason can be found in a higher deposition rate, which might influence the growth mechanism and the crystallinity.

While increasing the oxygen content, unexpectedly the transmittance is not improving (Figure S3), and the maximum reached value is 91.1% at a wavelength of 500 nm for the ITO film without oxygen gas introduction. Therefore, the ITO process without oxygen flow rate and with 40 sccm of argon gas was employed subsequently.

The previous ITO optimization was necessary to determine the RF power density and the number of cycles of the process. Nevertheless, the damages were still observed, and the typical *S*-shape appeared in the *J*–*V* curves (Figure S4), proving the drastic changes caused to the exposed layers of the devices.

In order to protect the substrates from sputtering damage, a TMO was employed as the PBL on top of PTAA prior to ITO sputtering. TMOs find application in sputtering damage prevention as well as in interface engineering for the enhancement of charge injection or extraction. TMOs, such as molybdenum oxide (MoO_*x*_), vanadium oxide (V_2_O_*x*_) and tungsten oxide (WO_*x*_), can be employed as hole-selective contacts due to their deep work function (Φ > 5 eV).^64−68^ TMOs’ low parasitic absorption is highly desired for applications in solar cells and optoelectronic devices. The main drawback of TMOs is the significant environmental sensitivity,^69^ which can influence the absorption as well,^50^ and can be mitigated with an encapsulation in inert atmosphere.

**Figure 2:**
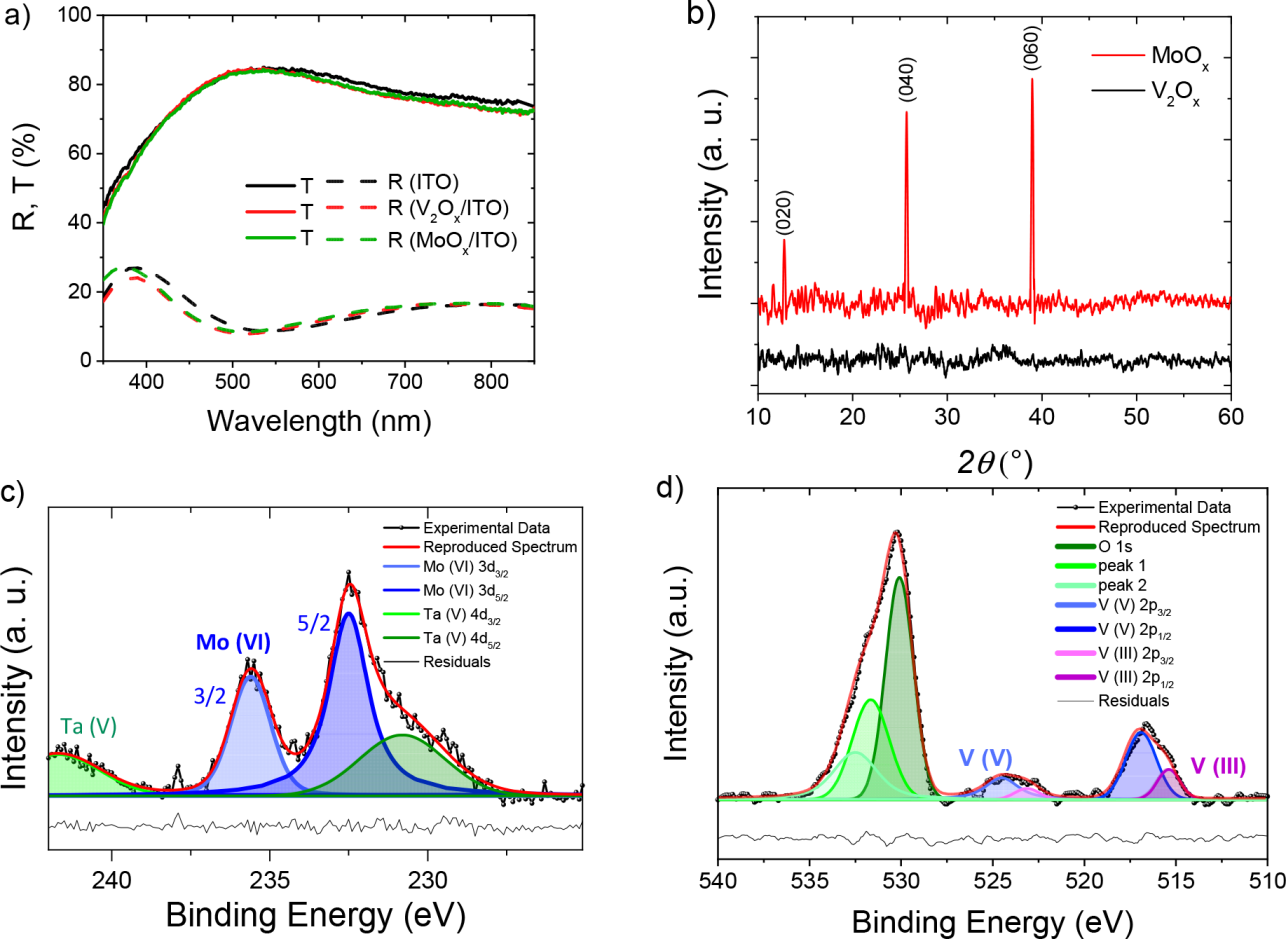
(a) Reflectance (dashed line) and transmittance (solid line) of ITO, V_2_O_*x*_/ITO and MoO_*x*_/ITO layers deposited on glass, from 350 to 850 nm. (b) XRD patterns of evaporated MoO_*x*_ and V_2_O_*x*_; c, d) XPS spectra of MoO_*x*_ buffer layer in the energy range of Mo 3d species (c) and of the sample with V_2_O_*x*_ atop in the corresponding energy range (d).

Here, we investigated two different TMOs (V_2_O_*x*_ or MoO_*x*_), as PBLs. The optical data of ITO on glass, with and without 5 nm of evaporated PBL (V_2_O_*x*_ or MoO_*x*_), were examined in order to analyze the possible additional parasitic absorption when introducing the PBL. The absorbance was calculated from the reflectance (*R*) and transmittance (*T*) data (Figure 2a), as 1– *R* – *T*, and is reported in Figure S5. A slight blue cutoff is shown in the absorbance spectrum of the V_2_O_*x*_/ITO layer, which was already observed in the literature.^50^ In our case, the shift is minor and negligible. An increased absorption is detected in the range between 380 and 520 nm, which can slightly impact the photocurrent generation of the device when illuminated from ITO-side and can be ignored for mechanically stacked tandem applications. From 750 nm on, the transmittance of the PBL/ITO layers is lower than the ITO transmittance. However, this is not affecting the device performance since it is only slightly overlapping with the absorption regime of perovskite (∼300–770 nm). The additional parasitic absorption losses were calculated for the MoO_*x*_/ITO and V_2_O_*x*_/ITO layer in comparison with ITO layer on glass for AM1.5G illumination, as shown in Figure S6. The cumulative current losses amount to ∼0.4 mA/cm^2^ for MoO_*x*_/ITO and ∼0.6 mA/cm^2^ for V_2_O_*x*_/ITO, which are negligible and are not impacting the photocurrent generation in the semitransparent solar cell.

The surface coverage and the film morphology of MoO_*x*_ and V_2_O_*x*_ were analyzed by energy-dispersive X-ray spectroscopy (EDX) elemental maps and atomic force microscopy (AFM). We observed a sufficiently uniform coverage in both films for the different analyzed thicknesses, even though a better uniformity was shown in MoO_*x*_ layer (see SI – Figure S7).

The crystallinity of the PBLs was studied with X-ray diffraction (XRD) analysis to reveal the morphology of the evaporated oxides. The MoO_*x*_ and V_2_O_*x*_ diffraction patterns are shown in Figure 2b. The peaks from the XRD pattern are assigned to the (020), (040) and (060) planes, which are attributed to the orthorhombic structure of MoO_3_.^70,71^ On the other hand, no peaks are detected in the spectrum of V_2_O_*x*_, revealing the amorphous structure of the evaporated V_2_O_*x*_. The morphology of the thermally evaporated TMOs can be correlated to the conductivity of the films. Thus, it can influence the charge carrier extraction when employing TMOs as PBLs in ST-PSCs.

It has been reported that oxygen vacancies play a crucial role in charge transport in TMOs. Oxygen deficiency can create gap defect states, which assist in charge transport. However, the work function increases with the oxygen content, which might influence the barrier height and the charge transfer at the interface.^64^ In other works, the formation of negative dipoles at the interface between TMO and the p-layer has been observed, which can promote the upbending of the p-layer. This can enhance charge extraction and, at the same time, create an electron barrier promoting separation of carriers.^66,72^ In order to deeply investigate the influence of oxygen vacancies on charge transport, we identified the oxidation states of the oxides by performing X-ray photoelectron spectroscopy (XPS). Shirley background subtraction was adopted, and the fitting process was done taking into account the Coster–Kronig effect.^73^ For MoO_*x*_, four different peaks are identified in the binding energy (BE) range of the Mo 3d core levels (Figure 2c). The spectrum is dominated by the doublet 3d_3/2_ and 3d_5/2_ caused by the spin–orbit splitting.^73,74^ The energy gap is 3.1 eV, with the major peak at 232.5 eV and the minor peak at 235.6 eV, in good agreement with values reported in literature.^75−79^ All of the fitting parameters are reported in Table S3. The corresponding oxidation state is the Mo (VI). Another small contribution is observed at lower energies (230.8 eV), which is associated with the Ta 4d_5/2_ spin–orbit component of the Ta oxide (Ta_2_O_5_). The 4d_3/2_ component is detected at higher energies, resulting in a spin–orbit splitting of 11 eV.^80,81^ This contribution is attributed to the sample holder used for the measurement. The absence of other oxidation states of molybdenum oxide at lower energies might limit the recombination at the PTAA interface and enhance hole extraction.^82,83^

For the vanadium oxide films, two species were discerned from the deconvolution process in the vanadium energy range (Figure 2d, with fitting parameters in Table S7). The main peaks of V 2p_3/2_ were found at 516.9 and 515.4 eV. The energy split Δ between the O 1s core level and the V 2p_3/2_ level was used for the oxidation states identification, as suggested in refs (84−86). In our case, the values of Δ are 13.2 eV for the main V 2p_3/2_ component and 14.7 for the other vanadium specie. Thus, the envelopes were associated with the oxidation states V (V) and V (III).^84−88^ The coexistence of two oxidation states discloses the oxygen deficiency of the film. In the spectrum, the oxygen peak is also present and the fitting process of O 1s spectrum discloses three peaks: the main oxygen peak at 530.1 eV and two lateral peaks at higher energies (531.6 and 532.5 eV), associated with oxygen ions and weakly adsorbed species, as for MoO_*x*_ (see SI).

Ultraviolet photoelectron spectroscopy (UPS) analysis was conducted on the oxides and the PTAA film in order to investigate the energy alignment. The work function (Φ) values were extracted from the UPS spectra (Figure S11) and are reported in Table S8. The work function of oxides (MoO_*x*_ and V_2_O_*x*_) might be influenced by air-exposure, and the measured values might be lower than expected.^66^ The difference Φ_TMO_ – Φ_PTAA_ is smaller in the case of MoO_*x*_, which might favor the ohmic contact formation. The aforementioned properties, such as the low parasitic absorption as well as the high work function and the analysis of oxygen vacancies, represent overall the characteristics for a suitable and promising hole-selective buffer layer in ST-PSCs.

**Figure 3:**
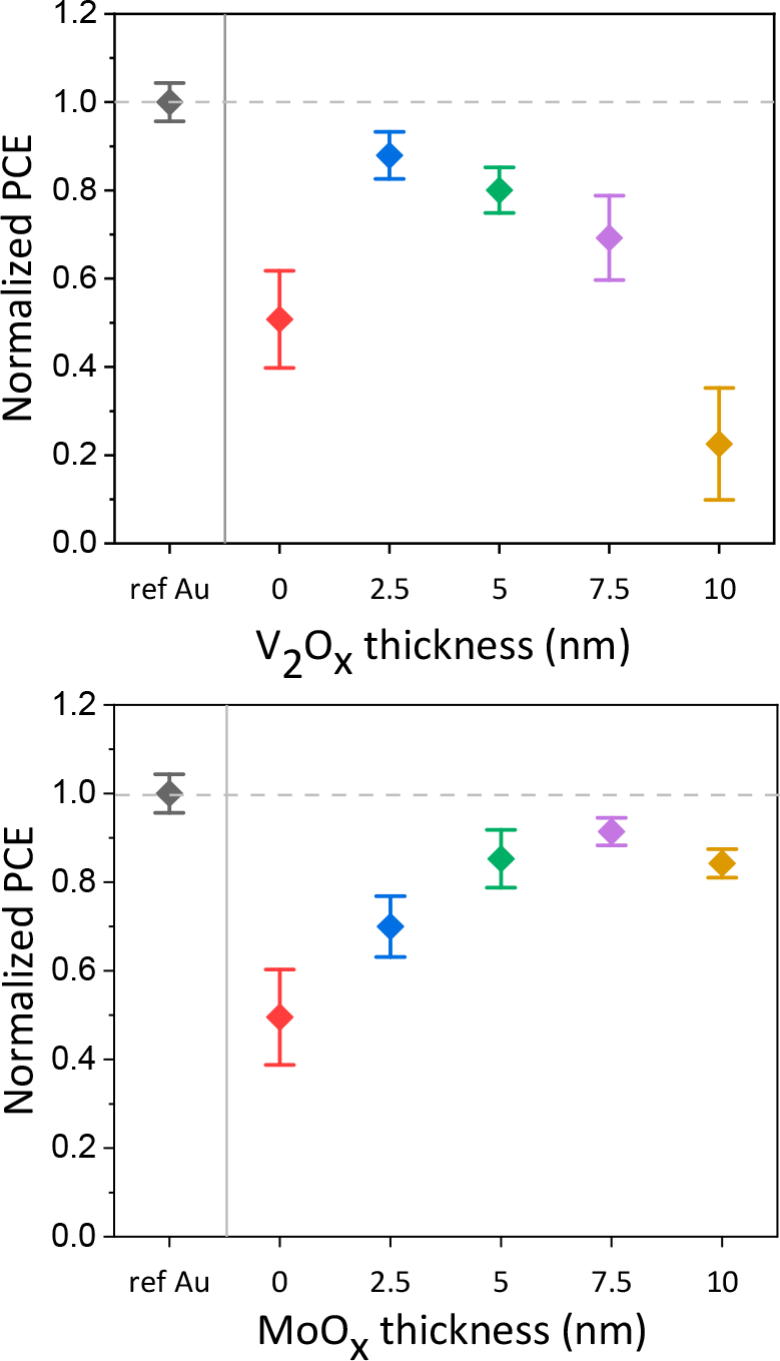
Normalized power conversion efficiencies (PCEs) of semitransparent perovskite solar cells under test. A range of thickness varying from 0 to 10 nm was analyzed for V_2_O_*x*_ (left side) and MoO_*x*_ (right side) as PBLs. The efficiencies of the semitransparent devices are compared with the corresponding opaque references (ref Au).

**Figure 4:**
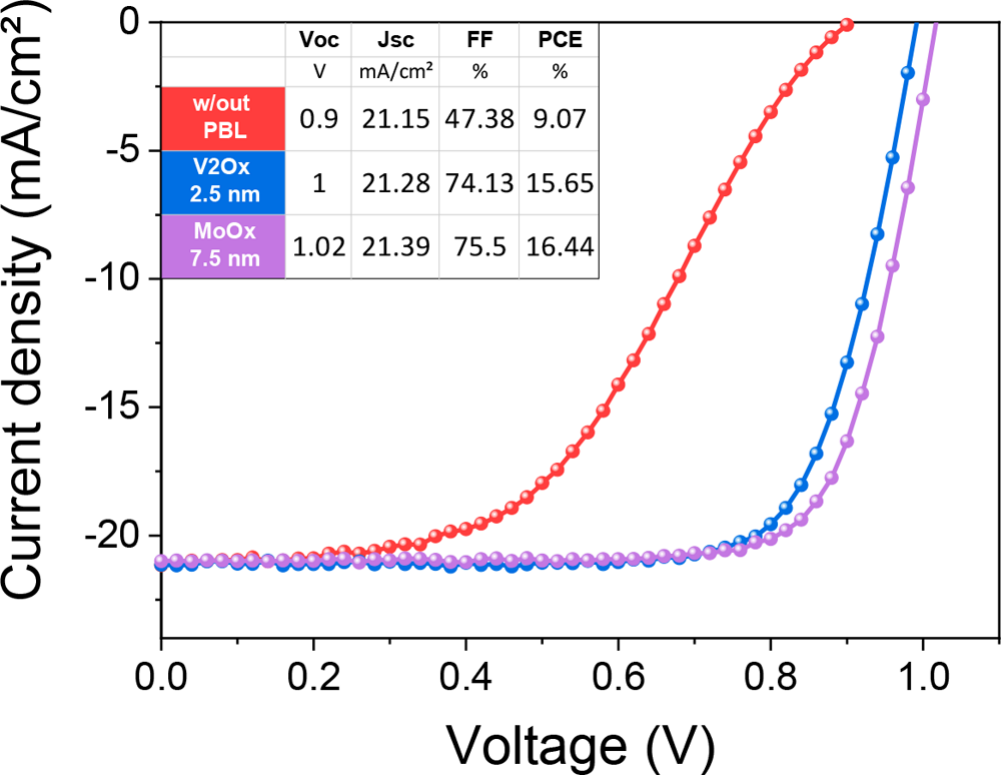
Current density–voltage (*J*–*V*) characteristics of the best devices with V_2_O_*x*_ and MoO_*x*_-based devices, compared with a device without PBL. In the table, the main electrical parameters of the cells are listed. The *J*_SC_ values are confirmed by the incident photon-to-current efficiency (IPCE) in Figure S15.

The impact of varying PBL thickness, ranging from 0 to 10 nm, on device performance was analyzed for the two types of oxides. Electrical parameters were compared with gold-based cells, which were used as reference samples and are reported in Table S9. These parameters were also normalized with the gold reference to facilitate a better comparison and are presented in Figure 3 and in Figure S12. The data from the gold-based references of the two batches are comparable since the error bars around the means overlap (Table S9), indicating no statistical difference between them. In the case of MoO_*x*_, all PBL-based devices exhibited higher FF and higher electrical performance compared to the devices without PBL. However, this was not the case for V_2_O_*x*_-based devices. Instead, an abrupt decay was observed when the PBL thickness was increased to 10 nm, mainly due to the decrease in *J*_SC_. The *J*_SC_ exhibited a pseudolinear trend up to 7.5 nm, with a reduction of approximately 0.29 mA/cm^2^ per nm. However, a sharp decrease was observed at 10 nm, which is likely due to a different film growth and stoichiometry with thickness. In the literature, an O/V ratio dependence with thickness has been reported.^89−91^ Gerling et al. demonstrated an excess of V^+4^ cations in thin V_2_O_*x*_ layers, which is attributed to oxygen deficiency, and an increase of V^+5^ species with the increase of the layer thickness, which might be proof of oxygen excess.^91^ Hence, while the lack of oxygen in thinner V_2_O_*x*_ layers might involve the formation of conductive states and enhancing carrier transport,^89^ a thicker V_2_O_*x*_ layer might reduce oxygen vacancies, thus diminishing electrical properties. Indeed, an increase of the series resistance (*R*_S_) and subsequent FF decay was also observed from 5 nm to higher thicknesses (Figure S12, Figure S13). The highest *V*_OC_s are observed for devices with 5 nm-thick V_2_O_*x*_ layer. A thicker layer might induce a stronger energy band bending in comparison to 2.5 nm-thick V_2_O_*x*_ layer and thus increase the *V*_OC_. The best device performance of 15.65% was reached with the thinnest V_2_O_*x*_ layer (Figure 4) with an FF of 74.13%. When using MoO_*x*_ as a PBL, the device performance was mainly influenced by the FF trend with the thickness. Current and voltage showed an almost constant behavior with the thickness variations (Figure S12). In the range 2.5 and 7.5 nm, the FF was increasing and the reason can be found in a stronger shield-effect to sputtering damage. However, for thicker layers, additional series resistances started to play a crucial role in FF, as shown in Figure S14. Therefore, a thickness of 7.5 nm was chosen, and the best device performance is 16.44% (Figure 4) with a superior FF of 76.22%. The slight overperformance of the MoO_*x*_-based devices might be justified with its crystalline morphology, which can enhance conductivity and charge carrier extraction, and with its better film coverage.

**Figure 5:**
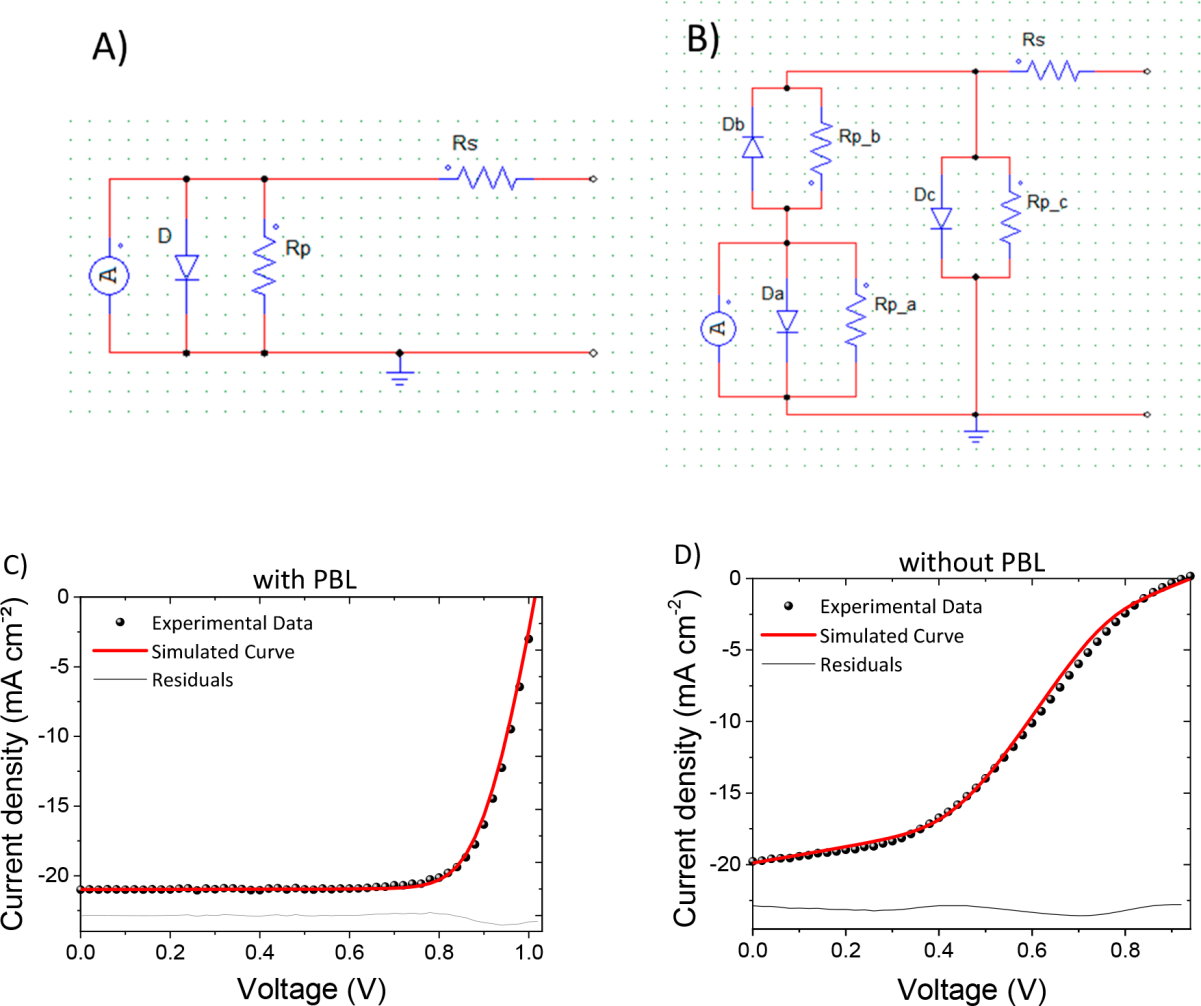
Equivalent circuit models used for the simulations of the device with (A) and without (B) PBL (B). Experimental and simulated current density–voltage curve of the device with PBL (C) and without PBL (D). The calculated residuals are also reported for each case.

In order to investigate the underlying phenomena of the *S*-shape and to gain a deeper understanding of the resultant damage to ITO sputtering, electrical simulations were conducted using the SPICE simulator software LTspice^92^ on devices with and without PBL. A MoO_*x*_-based device *J–V* curve was simulated, by using the standard equivalent circuit model of a solar cell (Figure 5A), and the resulting curve is well-aligned with experimental data (Figure 5C). For cells without PBL, a different circuit was employed, which accounted for the structural and electrical changes resulting from ITO sputtering and the subsequent *S*-shape in the *J–V* curve. The employed circuit was adapted from the work of Kanda et al.,^27^ with the addition of a shunt resistance (*R*_*P_C*_) in parallel to diode *D*_*C*_ to account for an additional leakage path between the solar cell terminals, as presented in Figure 5B. The diode *D*_*A*_ is modeling the ETL/perovskite/HTL layer stack and a slight increase of the ideality factor *n*_*A*_ from 1.5 to 1.6 is observed when sputtering directly on PTAA. Moreover, a parasitic series resistance was added to the diode (*R*_*S_A*_). These minimal electrical differences can be attributed to variation of the perovskite/PTAA interface upon ITO sputtering. An increase in series resistance *R*_*S*_ was observed as well. The diode *D*_*B*_ models the Schottky barrier increase at the PTAA/ITO interface and the changes in the work function upon ITO sputtering. In Kanda et al.’s work, the diode *D*_*C*_ is attributed to the physical damages of the HTL and is modeling the observed HTL shrinkages. Thus, in our case, the presence of diode *D*_*C*_ reflects the damage of the PTAA. Since all three diodes were modeled, the ITO sputtering is affecting the PTAA itself, as well as the PTAA/ITO and perovskite/PTAA interfaces. All of the parameters extrapolated from simulations can be found in Table S10. The presence of the *S*-shape that limits the efficiency of the device is also independent of the HTL thickness (Figure S16), which further demonstrates that the damages to the HTL and its interfaces rule the device performance.

**Figure 6:**
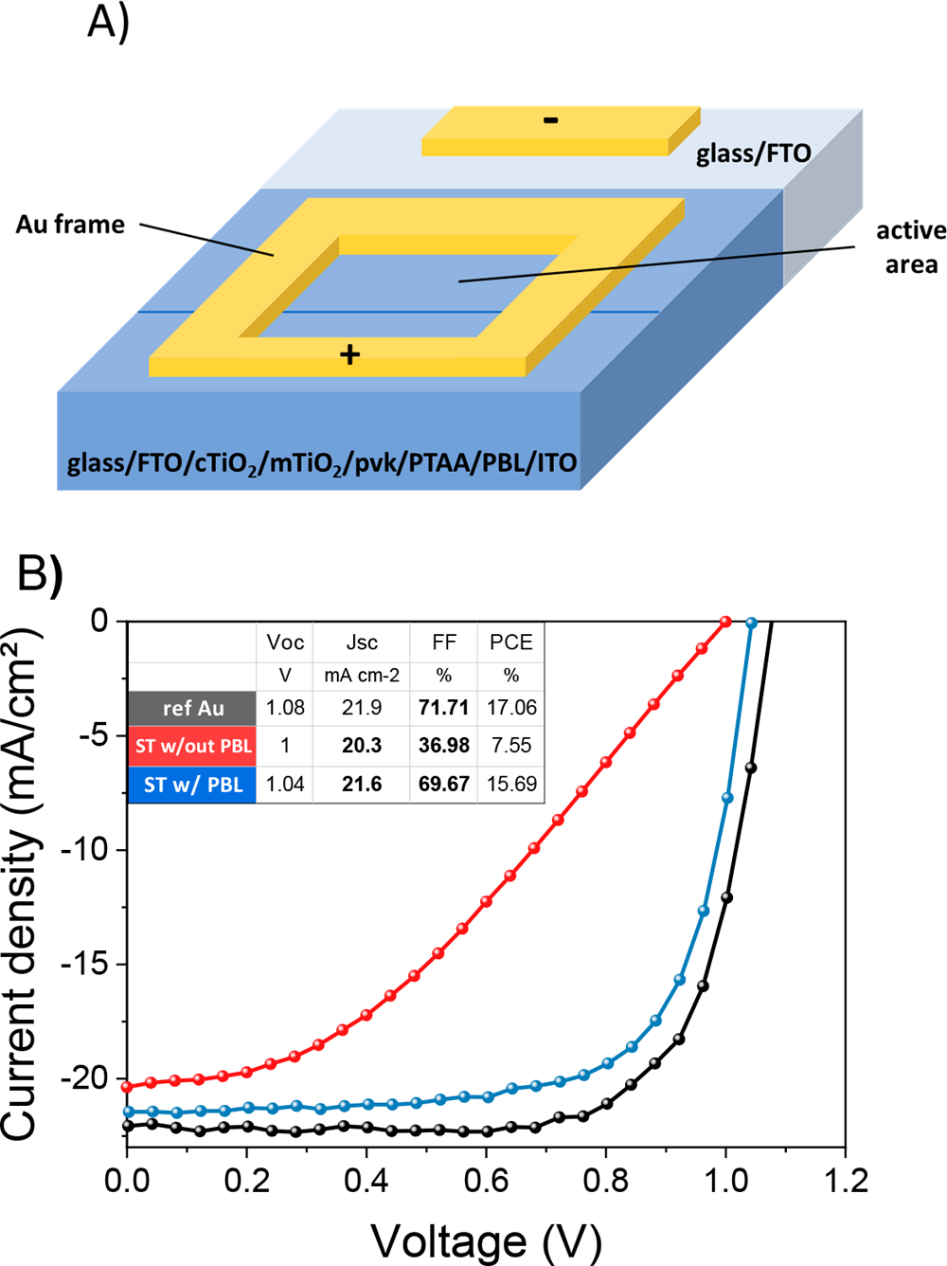
(A) Schematic of the layout of the large area ST-PSC with the gold frame. (B) Current density–voltage curve under AM 1.5G of the large area opaque cell (black curve) and ST-PSCs without PBL (red curve) and with PBL (blue curve).

**Figure 7:**
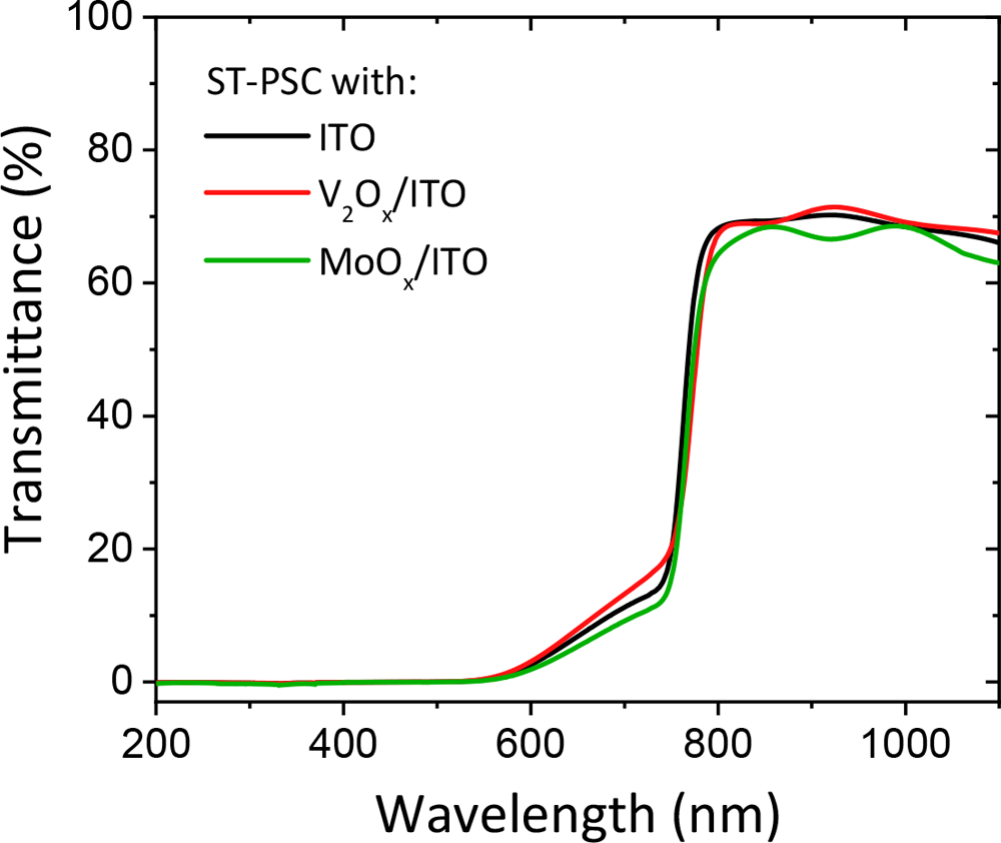
Transmittance of a complete semitransparent device without PBL (black curve) with V_2_O_*x*_ (red curve) or MoO_*x*_ (green curve) from 200 nm to the NIR (near-infrared, 1100 nm).

The upscaling process of ST-PSCs represents a necessary step to realize both semitransparent modules and tandem devices. Increasing the cell area usually implies an increment of the series resistance, as well as a reduction of the uniformity of all the deposited films.^93−95^ These issues dramatically affect the electrical performance of the upscaled devices. In this work, large area ST-PSCs (active area ≈1 cm^2^) were fabricated with MoO_*x*_ as PBL, and a gold frame was introduced to reduce the resistance losses and improve the FF, as shown in Figure 6A. The ultrathin PBL revealed to be efficient for sputtering damage prevention even in large area cells, since no *S*-shape was observed, as shown in Figure 6B. The introduction of the PBL increases the FF from 37% to approximately 70%. This will promote and facilitate the upscaling of semitransparent PSCs, and thus of BIPV and perovskite/silicon tandem. For the latter application, the transmittance spectra of complete devices with and without PBL were analyzed in the range from 200 to 1100 nm, as shown in Figure 7. The solar direct transmittance τ in the near-infrared (780–1200 nm) range is considered to investigate the effect of the PBLs in the wavelength range where silicon absorbs. τ is calculated as
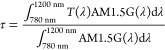
where *T*(λ) is the transmission spectrum and AM1.5G(λ) is the solar spectrum. The V_2_O_*x*_-based devices and the ST-PSCs without PBL exhibit the same τ value (68.6%), whereas a lower transmittance (66.4%) is obtained for MoO_*x*_-based devices. Thus, the effect of the PBL is negligible in the case of V_2_O_*x*_ since the devices showed the same solar direct transmittance percentage value of the devices without PBL. In the case of MoO_*x*_, a 3% reduction is observed. This might cause a loss in silicon absorption and reduce photocurrent generation in the silicon subcell in comparison with the V_2_O_*x*_-based ST-PSCs. In this work, FTO-coated glass substrates (Experimental Section) with a thickness of 2.2 mm were employed for device fabrication. However, a thinner substrate can be used to increase the transmittance in the NIR (see Figure S18), and thus, the absorption in the silicon subcell. An increase of τ to 72% is measured when employing a 1.7 mm-thick Pilkington FTO-coated glass substrate.

In the case of BIPV, the analogue parameter of τ in the visible range has to be considered, i.e., the average visible transmittance (AVT). A minimum value of 20% is required for solar window applications.^96^ Owing to the large panchromatic absorption of our perovskite (down to 780 nm), the AVT of our semitransparent devices is quite low (1.5%) resulting in an inadequate transparency for direct application in BIPV. A possible approach to restrain light absorption in the visible region is to decrease the perovskite thickness.^97,98^ Another option could be adopting a microstructure design and a patterned perovskite layer by introducing transparent regions.^99,100^ On the other hand, a different perovskite formulation with wider bandgap can be replaced in our device stack as an alternative approach.^101,102^ Here, we reduced the perovskite thickness from 500 to 50 nm by tuning the solution concentration from 1.4 to 0.4 M. However, for perovskite thicknesses lower than 300 nm, poor percolation of the perovskite within the mesoporous layer was observed. Thus, a tin oxide (SnO_2_) layer was deposited on top of c-TiO_2_ as substitute of m-TiO_2_ layer in devices with thinner perovskite.

**Figure 8:**
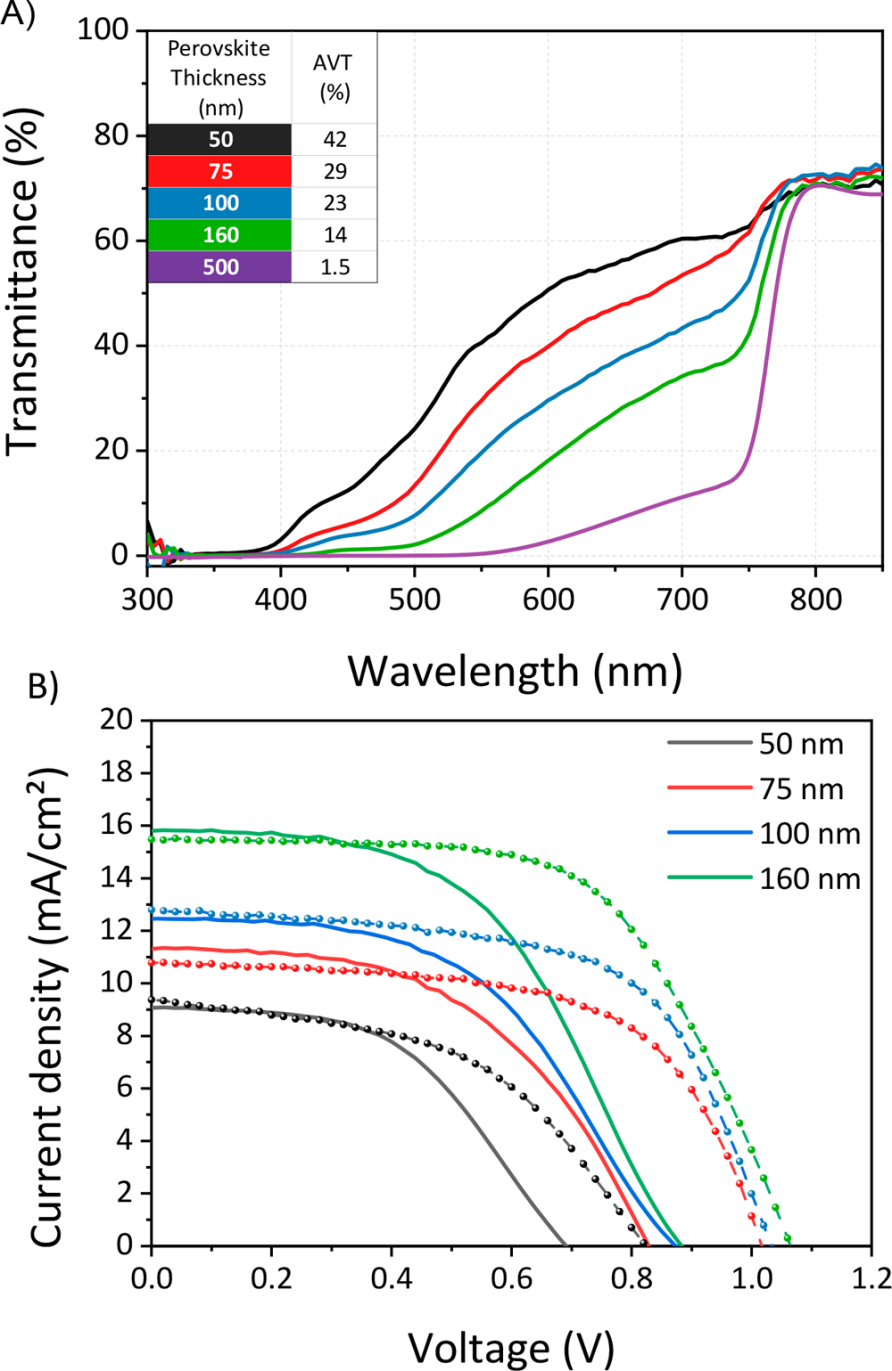
(A) Transmittance spectra of ST-PSCs with the structure glass/FTO/ETL/PVK/HTL/ITO with different perovskite thicknesses. The mesoscopic structure was employed for the device with 500 nm-thick perovskite. For lower perovskite thicknesses, the planar structure was used, and the m-TiO_2_ film was replaced with a SnO_2_ layer. (B) Current density–voltage curves of ST-PSCs with different perovskite thicknesses (reported in the legend) without (solid line) and with (dashed line and symbol) PBL. The electrical parameters can be found in Table S11.

As expected, a higher transmittance in the visible range was observed with a decrease of the absorber thickness (see Figure 8A). Thus, an improved AVT up to 42% was reached for lower perovskite thicknesses (Figure S19). As shown in Figure 8B, we found the typical *S*-shape in the *J–V* characteristics when the TCO was deposited directly on the HTL without PBL. The introduction of 2.5 nm of V_2_O_*x*_ (Figure 8B) successfully mitigated the *S*-shape by improving, at the same time, the open circuit voltage of ∼15–20 mV. Additionally, a comparable AVT is measured for devices with and without PBL to further prove that the PBL is introducing negligible parasitic absorption in the visible, as already shown in Figure S5 and Figure S6). These results clearly demonstrate the effectiveness of the PBL also for thin perovskite-based devices for BIPV applications.

To investigate the stability of the semitransparent PSCs and the effect of TMOs in the cell stack, a light soaking test (ISOS-L-1^103^) was performed. In the literature, Sanheira and co-workers demonstrated an improved stability of MoO_*x*_/Al-based devices. The explanation was found in the formation of an Al_2_O_3_ barrier that prevents iodization of the back contact and limits moisture-induced degradation. However, the test was performed under low relative humidity (RH < 20%) combined with the control of devices temperature at 30 °C.^104^ A completely different behavior of MoO_*x*_ is observed at temperatures relevant to PSCs operational condition. The direct contact between perovskite and MoO_*x*_ was analyzed by Schulz and co-workers.^105^ Lead and iodine oxidations, in combination with molybdenum reduction, create defect states at the perovskite/MoO_*x*_ interface, which was revealed to be detrimental for device functionality. Thus, an organic HTL (spiro-OMeTAD) buffer layer was necessary. The interaction of MoO_*x*_ and the organic HTL was explored by Schloemer and co-workers.^106^ In this study, the strong sensitivity of MoO_*x*_ on the growth surface of the spiro-OMeTAD was disclosed. The presence of nanoscopic pores in the HTL could induce buckling and subsequent delamination of MoO_*x*_ at operating temperatures. On the other hand, V_2_O_*x*_ was revealed to be morphologically more robust and stable on top of the organic HTL, due to its higher crystallization temperature (*T*_cryst_ = 600 °C).^106−108^ In addition to limiting moisture and oxygen ingress, V_2_O_*x*_ was shown to suppress the oxidation and the diffusion of Li ions, which are present in the doped organic HTL. Moreover, in opaque cells the vanadium oxide layer is preserving perovskite stability by hindering the migration of metal ions.^107^ In ST-PSCs, the instability of MoO_*x*_ has been assessed as well.^109^ Effective approaches that have been pursued are to either substitute it with ALD-V_2_O_*x*_^39^ or to introduce a spinned oxide nanoparticles-based layer prior to MoO_*x*_ deposition.^109^

**Figure 9:**
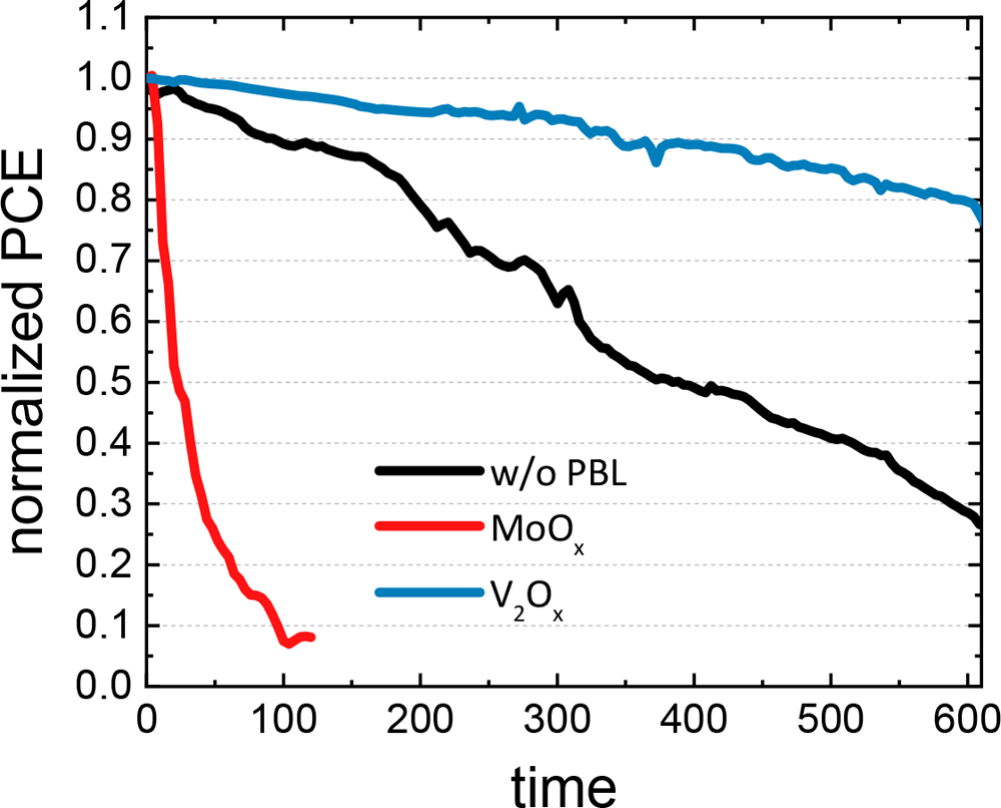
Normalized PCE over time of the semitransparent PSCs with and without PBL.

The stability of our devices was examined under operative conditions (maximum power point tracking, MPPT) in ambient atmosphere (i.e., ISOS-L-1). For this purpose, large area ST-PSCs with PBL (MoO_*x*_ or V_2_O_*x*_) and without PBL were encapsulated prior to stress test as reported in the Experimental Section. The normalized PCEs are shown in Figure 9, and the other electrical parameters can be found in the SI (Figure S21). When the PBL is not employed, the device reaches the *T*_80_ after about 310 h. As expected, a rapid decay of the PCE is observed in the MoO_*x*_-based ST-PSC and *T*_80_ was reached after 12 h of stress. As already established in the literature, this degradation can be attributed to the reduction of Mo at the interface with the perovskite/PTAA layers.^104,105,110^ Another explanation could be found in the possible delamination of MoO_*x*_ atop the organic HTL.^107^ On the other hand, evaporated V_2_O_*x*_-based ST-PSCs showed a superior stability compared to the other devices under test with a *T*_80_ equal to 600 h, confirming its morphological robustness and outstanding chemical inertness.^105−108^

## Conclusions

This work presents an optimization of the ITO top electrode deposition and the prevention from sputtering damage in n-i-p ST-PSCs, with the aim of developing these devices for tandem applications and see-through PV. Electrical simulations were performed on sputtering-damaged devices, revealing that the ITO sputtering affects the PTAA layer as well as the related interfaces, i.e., perovskite/PTAA and PTAA/ITO. The ITO deposition process was optimized, balancing the UV plasma effect and the ion bombardment effect, and complete suppression of sputtering damage was achieved with the introduction of an evaporated TMO atop the PTAA layer. The use of ultrathin PBLs (2.5 nm for V_2_O_*x*_ or 7.5 nm for MoO_*x*_) effectively mitigated the sputtering damage effects. The introduction of the PBL (either MoO_*x*_ or V_2_O_*x*_) eliminated the *S*-shape and mitigated the reduction of the efficiency that characterizes the ST-PSCs in comparison with the gold-based PSCs, by reaching 88% for the V_2_O_*x*_ (PCE = 15.65%) and 91% for MoO_*x*_ (PCE = 16.44%) of the performance of the gold-based devices. The amorphous structure of the evaporated vanadium oxide might explain the slight underperformance of the V_2_O_*x*_-based ST-PSCs. Moreover, UPS disclosed a higher work function difference Φ_TMO_ – Φ_PTAA_ in the case of V_2_O_*x*_, which can justify the lower detected *V*_OC_ values.

In our study, we successfully demonstrated that the PBL is effective at suppressing the sputtering damage also in thinner perovskite-based ST-PSCs for BIPV application, without impairing the AVT. Additionally, we upscaled the ST-PSCs up to 1 cm^2^ and confirmed that the PBL effectively prevented sputtering damage in large area cells. Furthermore, the V_2_O_*x*_-based ST-PSCs demonstrated superior stability during the light soaking stress with a *T*_80_ equal to 600 h, while the MoO_*x*_-based devices only reached *T*_80_ after 12 h. Thus, even though the ST-PSC with ultrathin V_2_O_*x*_ PBL exhibited a slightly reduced efficiency compared to those with MoO_*x*_, the superior transmittance in the NIR and higher stability of the former oxide make it a promising PBL candidate for preventing the ITO sputtering damages in ST-PSCs for perovskite/silicon tandem or BIPV applications.

## Experimental Section

### Device Fabrication

PSCs were fabricated on commercial fluorine-doped tin oxide (FTO) coated glass substrates (Pilkington TEC7, 2.2 mm thick, 7 Ω sq^–1^). The glass/FTO sheets were patterned with a nanosecond raster scanning laser (λ = 1064 nm, Nd:YVO_4_) and then cut in 2.5 × 2.5 cm^2^ samples. The FTO-patterned samples were cleaned in ultrasonic bath with a 2% solution of Hellmanex detergent in deionized water, acetone, and then isopropanol for 10–15 min. Any remaining solvent residual was blown off using an air flow. UV-ozone treatment was then performed on the substrates for 15 min to remove all the residual organic contaminants with a PSD Pro Series Digital UV Ozone System from “Novascan”.

The compact TiO_2_ layer was deposited by manual spray pyrolysis on the substates from a precursor solution of 0.5 mL of acetylacetone (from Merck) and 0.75 mL of titanium(IV) diisopropoxide bis(acetylacetonate) (from Sigma-Aldrich) in 11.25 mL of ethanol (EtOH). The substrates were first left for 10 min on a hot plate at 460 °C, and then the deposition was performed with the nozzle about 40 cm far from the FTO surface and with an angle of about 45° with respect to the substrate plane. The spray gun was following a serpentine path for 12 cycles (one every 10 seconds). Then, the substrates were left for 15 min on the hot plate at 460 °C before cooling them down slowly to room temperature.

A solution composed of anatase titania nanoparticles paste (30NR-D, GreatCell Solar) diluted with EtOH, (w/w ratio of 1:5) was spin coated on the c-TiO_2_ surfaces at 3200 rpm for 20 s and annealed at 120 °C for 15 min. A sintering process was then performed as reported in.^111^ Alternatively, a solution of SnO_2_ (tin(IV) oxide, 15% in H_2_O colloidal dispersion) diluted with deionized water (v/v ratio of 1:20) was spin coated at 3000 rpm for 25 s, where specified in the paper. A subsequent annealing step of 20 min at 120 °C was performed.

The perovskite solution was prepared using PbI_2_ and PbBr_2_ purchased from TCI, FAI, MAI and MABr from GreatCell Solar, and the N–N dimethylformamide (DMF) and dimethyl sulfoxide (DMSO) from Sigma-Aldrich. The perovskite composition of Cs_0.08_ FA_0.80_ MA_0.12_ Pb (I_0.88_ Br_0.12_)_3_ was obtained by preparing a solution containing FAI (1 M), PbI_2_ (1.2 M), PbBr_2_ (0.2 M), CsI (0.1 M), and MABr (0.2 M), diluted in DMF and DMSO in a volume ratio of 1:4. It was stirred overnight and spin coated in a glovebox in a two-step protocol, 2000 rpm for 10 s and then 5000 for 30 s,^111^ and the chlorobenzene as antisolvent was dropped during the second step. The samples were annealed at 100 °C for 1 h.

After heat treatment, the substrates were cooled and the PTAA layer was deposited. For PTAA solution preparation, PTAA powder from Solaris Chem (SOL2426L, low molecular weight) was diluted in toluene (Sigma-Aldrich) with a concentration of 10 mg/mL. The solution was then doped with lithium bis(trifluoromethanesulfonyl)imide (Li-TFSI) of stock solution (520 mg in 1 mL of acetonitrile) and 4-*tert*-butylpyridine (TBP). The opaque cells were terminated with a 100 nm-thick evaporated gold contact atop the PTAA layer. For the ST-PSCs with PBL, either molybdenum(VI) oxide powder or vanadium(IV) oxide powder (from Sigma-Aldrich) was thermally evaporated with a rate of 0.1 Å/s. A linear radio frequency (RF) sputtering system from Kenosistec was employed for indium tin oxide (ITO) deposition from a target with a In_2_O_3_/SnO_2_ composition of 90:10 wt %. A base pressure of 5 × 10^–6^ mbar was reached before starting a presputtering step in order to remove target impurities and to improve reproducibility of the process. The ITO deposition was carried out with a working pressure of 1.1 × 10^–3^ mbar. A horizontal motion of the substrate holder was performed to increase the film uniformity. The argon and oxygen flow rates were tuned as described in the Results and Discussion section. Ar and O_2_ were introduced from two different specific tanks. The input power density was varied from 0.26 to 0.52 W/cm^2^, as well as the number of sputtering cycles, in order to deposit always a 100 nm-thick ITO film.

Before the light soaking stress test (ISOS-L-1), the devices were encapsulated with a thermally curable commercial adhesive polymer/resin (31X-167-2D ThreeBond). The material was laminated on top of the devices with the industrial laminator Core Model 2 Automatic Solar Panel Laminator by Rise Technology srl. The encapsulation process was controlled in temperature (*T* = 60 °C for 10 min) and pressure (between 600 and 700 mbar, during the plateau at 60 °C of temperature ramp).

### Characterization

*J*–*V* measurements of the PSCs were performed with a Class-A Sun Simulator (ABET 2000) equipped with an AM1.5G filter (ABET). The calibration of the Sun Simulator was made by using a Si-based reference cell (RR-226-O, RERA Solutions) to obtain a 1 Sun Illumination Condition. Arkeo platform (Cicci Research s.r.l.) was used for *J*–*V* characterization under forward and reverse scan direction and for MPPT. A voltage step of 20 mV/s and a scan rate of 200 mV/s were set.

EQE characterization was performed with an Arkeo system (Cicci Research s.r.l.) with a 150 W xenon lamp and a double grating (300 to 1400 nm). A Si photodiode was used for incident light calibration prior to the EQE measurement.

An UV–vis spectrophotometer (Shimadzu UV-2550) equipped with an integrated sphere was used for the acquisition of transmittance and reflectance spectra. The spectra from 200 nm to the NIR region were acquired with an UV–vis-NIR Jasco V-630 Double Beam Spectrophotometer equipped with a single monochromator. The sheet-resistance of the sputtered ITO was measured using a four-probe measurement setup and a source meter (Keithley 2620).

AFM measurements were performed with the microscope working in the repulsive regime of contact mode in air at room temperature. The Bruker silicon nitride MSNL-10 cantilevers were employed. Constant force images with a force of 1 nN were acquired with a typical scan rate of 2–4 s/row. The data were then analyzed using Gwyddion software. The energy-dispersive X-ray spectroscopy (EDX) elemental maps were acquired with an Xplore detector by Oxford Instruments.

X-ray diffraction (XRD) measurements were collected with a Rigaku SmartLab SE 1D Type diffractometer working in Bragg–Brentano geometry equipped with a Cu Kα source and a D/teX Ultra 250 detector.

X-ray Photoelectron Spectroscopy (XPS) spectra were recorded in a Vacuum Generators VG-450 ultrahigh-vacuum (UHV) chamber equipped with an Al Kα radiation source. The position of the Fermi level (E_F_) was determined with an accuracy of 50 meV by the photoemission from the metallic sample holder.

The ISOS-L-1 tests were performed with an Arkeo-multichannel (Cicci Research company) based on 32 fully independent Source Meter Unit (± 10 V @ ±250 mA) and an ARKEO Light soaker (VIS version) with low mismatch LED based system (400–750 nm). A standard Perturb & Observe tracking algorithm was selected with a *J*–*V* scan every 4 h. The measurements were done in ambient conditions without temperature control, and the cells were encapsulated prior to the test.

## Supporting Information Available

Transmittance and sheet resistance of ITO; Surface coverage and film morphology analysis; XPS and UPS spectra of MoO_*x*_ and V_2_O_*x*_ layers; Electrical parameters (*J*_SC_, *V*_OC_, FF), *J*–*V* curves, IPCE and MPPT of the examined ST-PSCs; Optical measurements and transmittance of the devices; AVT of the devices for BIPV applications; Electrical parameters of ST-PSCs under light soaking (PDF)
