# Supplementary material for: Semitransparent Perovskite Solar Cells with Ultrathin Protective Buffer Layers
Source: ACS Appl Energy Mater. 2023 Oct 5;6(20):10340–53. doi: 10.1021/acsaem.3c00735 (PMC10598631; doi:10.1021/acsaem.3c00735)
Supplement: Supplementary file 1 — ae3c00735_si_001.pdf [file ae3c00735_si_001.pdf]

Supporting Information

Semi-Transparent Perovskite Solar Cells with Ultrathin Protective Buffer Layers

Erica Magliano[1], Paolo Mariani[1], Antonio Agresti[1], Sara Pescetelli[1], Fabio Matteocci[1], Babak Taheri[2], Antonio Cricenti[3], Marco Luce[3] and Aldo Di Carlo[1,3]*

[1]C.H.O.S.E. (Center for Hybrid and Organic Solar Energy), Electronic Engineering Department, University of Rome Tor Vergata, Via del Politecnico 1, 00133, Rome, Italy.

[2]ENEA - Centro Ricerche Frascati, Via Enrico Fermi, 45, 00044, Frascati (Rome), Italy.

[3]Istituto di Struttura della Materia (CNR-ISM) National Research Council, via del Fosso del Cavaliere 100, 00133, Rome, Italy.

Corresponding author: aldo.dicarlo@uniroma2.it

ITO process	Sheet resistance [Ω/□]
0.26 W/cm^2_300 cycles	32 ± 4 m
0.34 W/cm^2_250 cycles	33.4 ± 4 m
0.39 W/cm^2_200 cycles	34.1 ± 4 m
0.45 W/cm^2_170 cycles	29 ± 3.6 m
0.52 W/cm^2_150 cycles	28.5 ± 3.5 m

Table S1: Sheet resistance for each type of ITO film deposited on glass. The sheet resistance was measured with the four-point probe method. The measured value and the accuracy are reported.

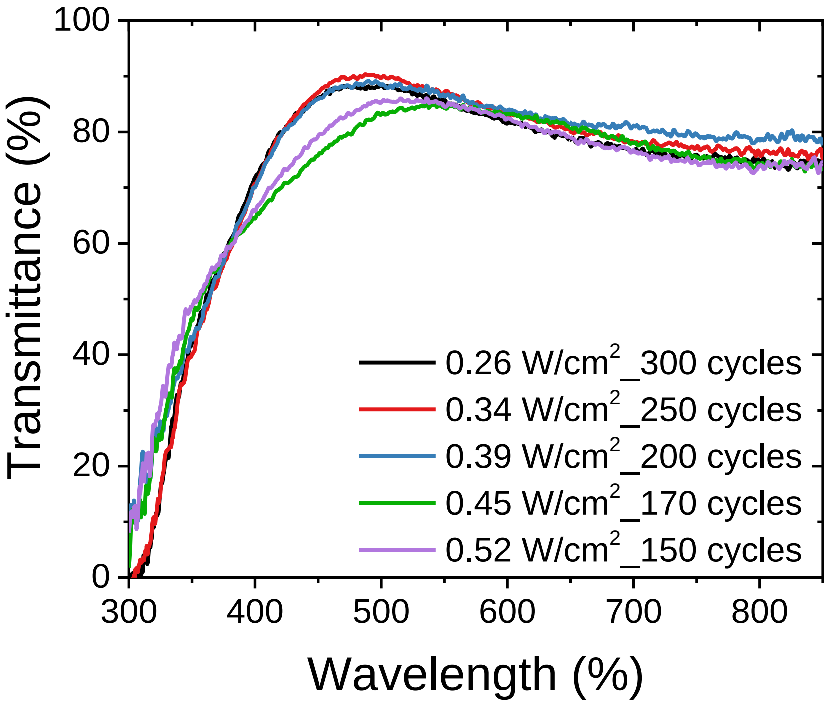

Figure S1: Transmittance spectra of each type of ITO film under examination

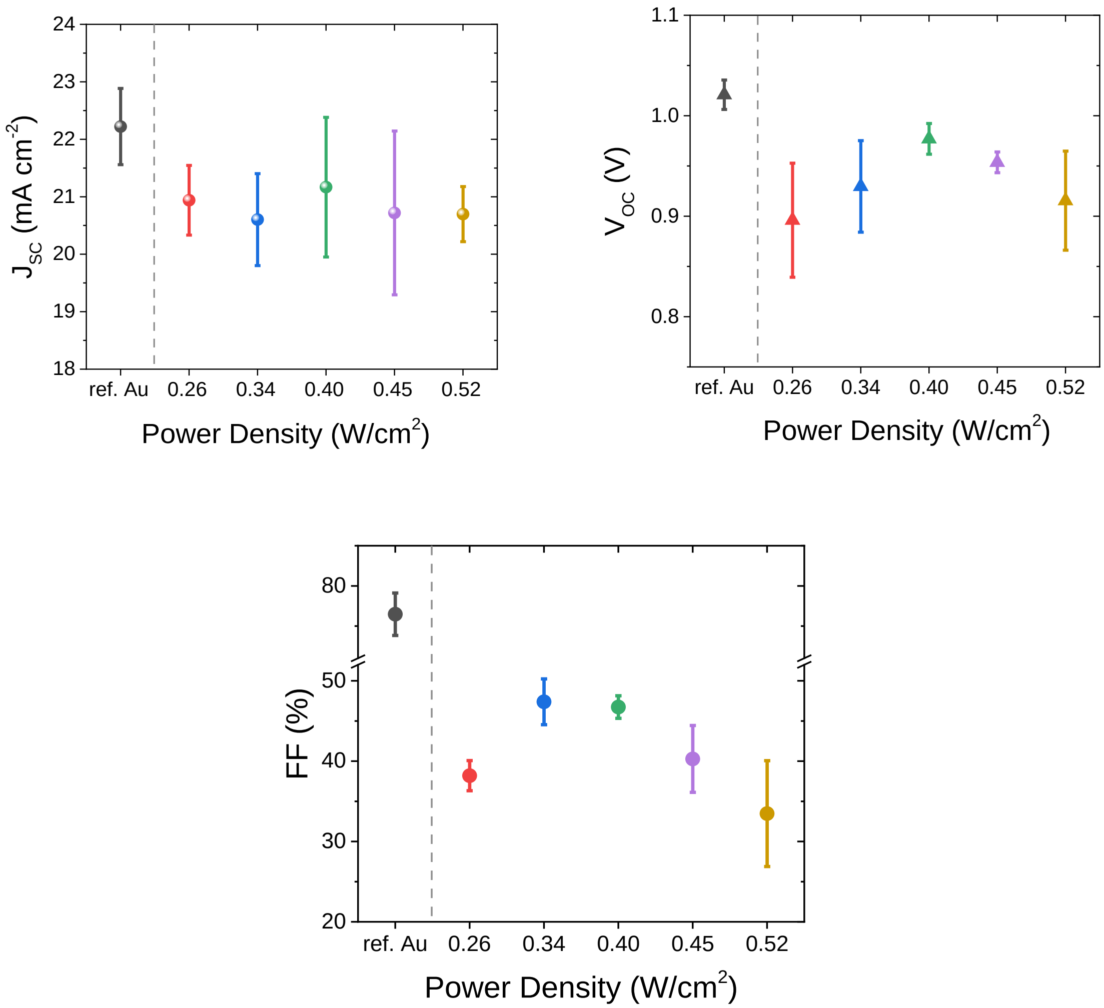

Figure S2: Electrical parameters (J_{SC}, V_{OC}, FF) as a function of the set RF power density of the sputtering system.

ITO process	Oxygen percentage (%)	Ar flow rate (sccm)	O_2 flow rate (sccm)	Sheet resistance [Ω/□]
**Ctrl ITO**	0	40	0	33
**ITO_1%**	1	40	0.4	1.7 k
**ITO_1.5%**	1.5	40	0.6	17 k
**ITO_2.5%**	2.5	40	1	23 k
**ITO_4.8%**	4.8	40	2	1 M
**ITO_0.5%**	0.5	80	0.4	12 K

Table S2: Oxygen and Argon concentrations are listed for each ITO sputter deposition that was tested. The sheet resistance was measured for each layer (glass/ITO) and is reported in the last column.

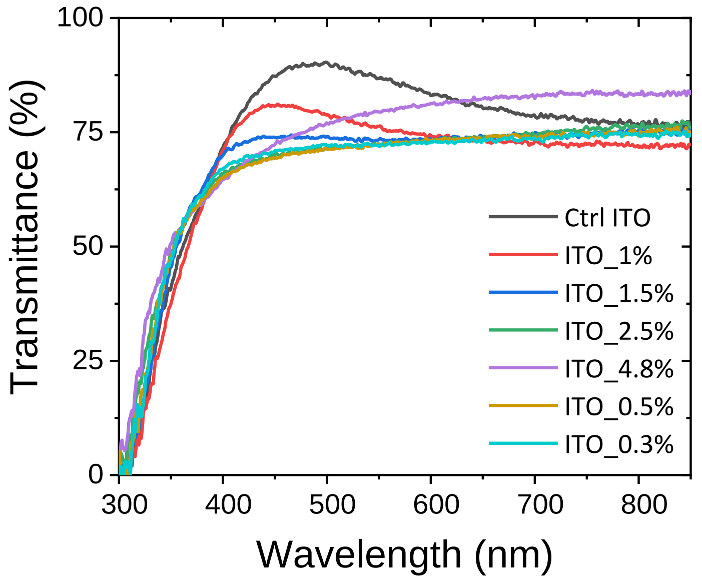

Figure S3: Transmittance as a function of wavelength from 300 nm to 850 nm of glass/ITO. The layers differ for the set oxygen flow rate during the sputtering process.

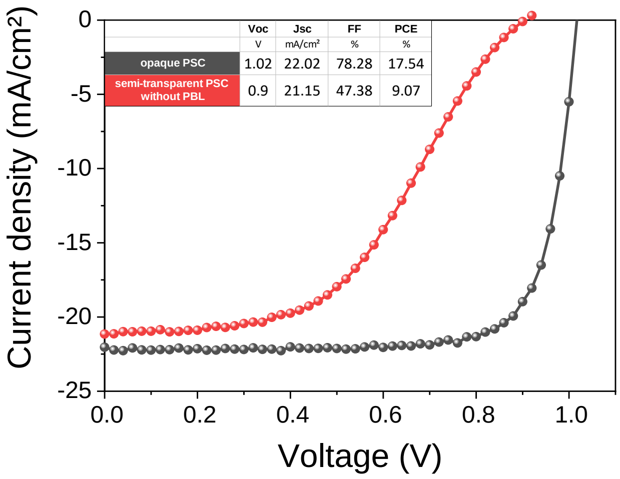

Figure S4: J-V characteristic of the gold-based opaque cell (black curve) and the semitransparent without PBL (red curve).

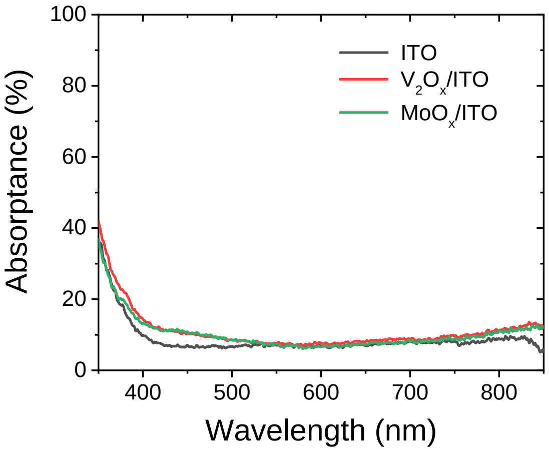

Figure S5: Calculated absorptance of ITO, V_2O_x/ITO and MoO_x/ITO layers deposited on glass.

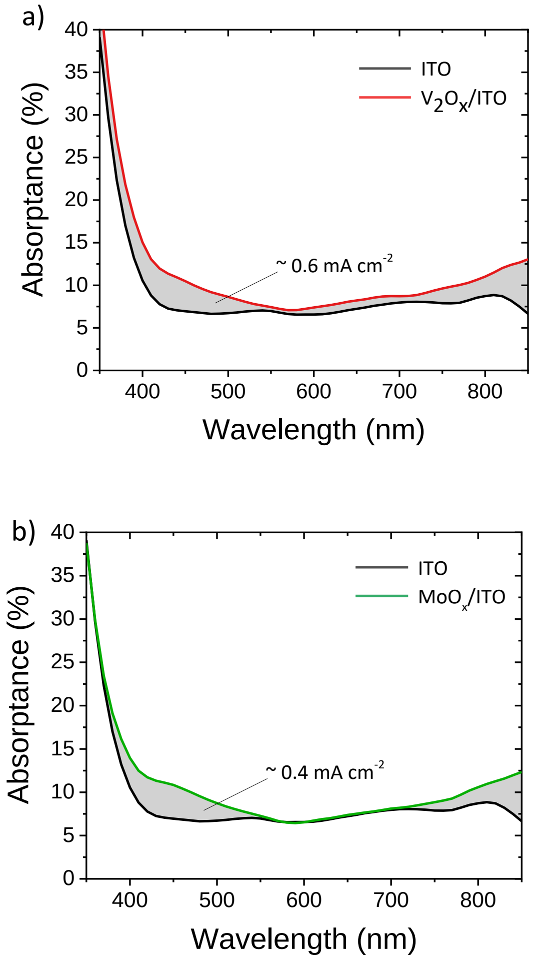

Figure S6: a, b) Absorbance of V_2O_x/ITO and MoO_x/ITO, respectively, and ITO. The grey areas represent the corresponding cumulative current losses.

The surface coverage of the V_2O_x films was examined through EDX elemental maps on glass/ITO/V_2O_x samples with two different thicknesses (2.5 nm, 10 nm) of the TMO layer. The analysis showed a similar surface coverage percentage of the film, from 53% of 2.5 nm-thick V_2O_x (Fig. S7 – B, D) to 58% of 10 nm-thick V_2O_x. Moreover, AFM measurement disclosed a quite similar morphology between 10 nm- and 2.5 nm-thick V_2O_x layers, as shown in Fig. S7 – A, C. In the first case, a continuous film with a surface rms roughness, σ_{RMS}, of 3.7 nm was observed, whereas for 2.5 nm-thick V_2O_x we observed a slightly more uneven structure, even though σ_{RMS} is 3.1 nm. However, a more irregular surface is not detrimental to the devices as regards the sputtering damage. Due to the presence of the heavy metal, the V_2O_x is still maintaining the intrinsic property of acting as a shield against ions (such as Ar^+, Ar, O^- and O_2^-) and X-rays present in the plasma during the sputtering process.[1,2] Nevertheless, the possible presence of cavities can be the cause of the observed V_{OC} reduction for thin layers since the PTAA/ITO interface can play a role in the energy band alignment.

In the case of MoO_x, a higher surface coverage of ~90% was detected with EDX analyses in 7.5 nm- and 10 nm-thick layers. The surface roughness of the films is approximately 4.5 nm in both cases, meaning that a continuous layer is deposited.

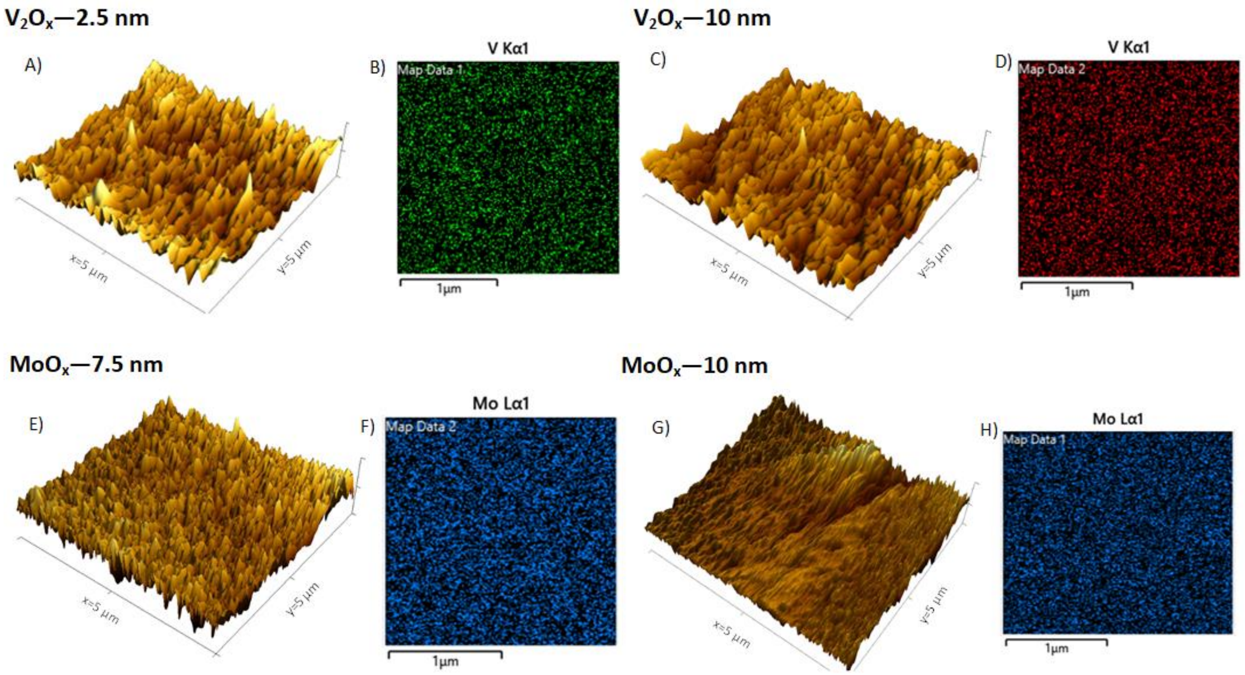

Figure S7: 3D AFM height images and EDX elemental maps of vanadium oxide and molybdenum oxide on glass/ITO: on the top, 2.5 nm-thick V_2O_x (A, B) and 10 nm-thick V_2O_x (C, D); on the bottom, 7.5 nm-thick MoO_x (E, F) and 10 nm-thick MoO_x (G, H).

	Peak Type	Area Fit	Center Max	FWHM
Mo 3d3/2	Voigt	145.6	235.6	1.5
Mo 3d5/2	Voigt	280	232.5	1.5
Ta 4d3/2	Gaussian	98.0	241.7	3.2
Ta 4d5/2	Gaussian	144.4	230.8	3.2

Table S3: Spectral fitting parameters of Mo 3d and Ta 4d species.

The range of BEs between 420 and 390 eV was also analyzed (Figure S8) and Mo 3p core levels were identified [3,4]. The peak energies are localized at 398.0 eV and 415.6 eV for the spin-orbit doublet $3p_{3/2}$ and $3p_{1/2}$, respectively. Other peaks in this BE interval were detected, which are likely to be due to N and Pb traces in the bulk. The presence of lead was also confirmed from the peak found at 20.6 eV (Figure S8, with fitting parameters in Table S5).

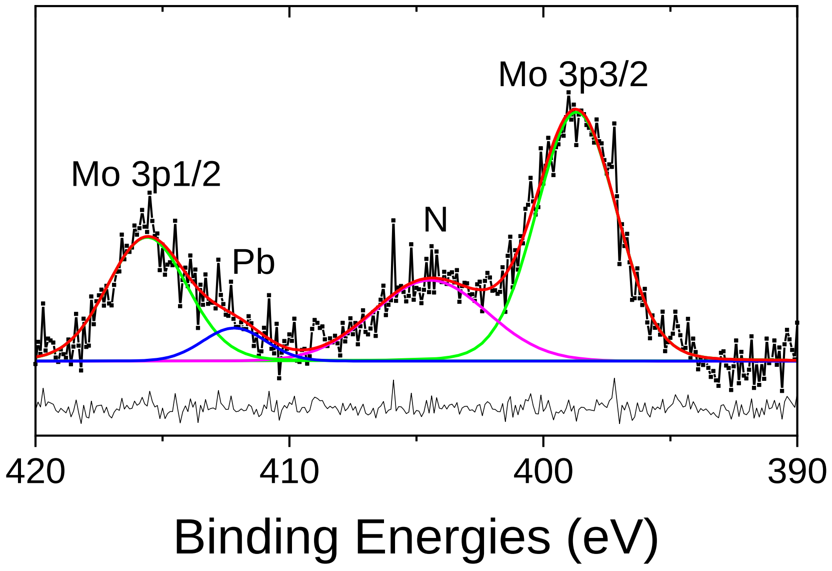

Figure S8: XPS spectra of MoO_x film in the energy range 390-420 eV.

	Peak Type	Area Intg	FWHM	Max Height	Center Grvty
N	Voigt	175.78734	5.02351	24.91092	404.52328
Mo 3p1/2	Voigt	132.00215	3.7168	33.1371	415.59562
Pb	Gaussian	13.40102	1.79199	7.02537	412.77831
Mo 3p3/2	Voigt	268.80547	3.7168	67.09584	398.67697

Table S4: Spectral fitting parameters associated to the spectra in Figure S7.

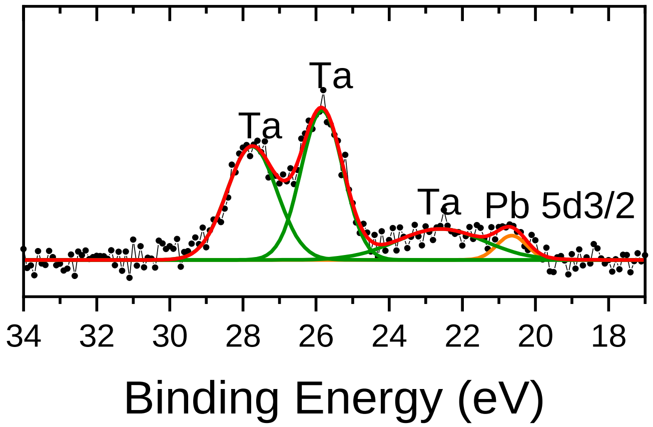

Figure S9: XPS spectra of MoO_x film in the BEs range 17-34 eV.

	Peak Type	Area Intg	FWHM	Max Height	Center Grvty
Pb	Gaussian	20.12952	0.96226	19.65215	20.6487
Ta	Gaussian	173.98384	1.35212	120.88226	25.83735
	Gaussian	157.63235	1.61324	91.79412	27.75253
	Gaussian	75.89593	2.84502	25.06117	22.59756

Table S5: Spectral fitting parameters associated to the spectra in Figure S9.

The O 1s spectrum is dominated by a main peak at 530.5 eV, which corresponds to crystal bulk oxygens [5,6]. Other components are detected at lower and higher energies in this energy interval.

In the O 1s spectrum of Transition Metal Oxides (TMOs), typically, two components can be discerned in the BE range 529.5-533. [7] The main peak is usually found in the BEs range 529.5-530.5 eV, which is ascribed to O^{2-} ions of the crystalline network. Moreover, a lateral structure is also present at higher binding energies (531-533 eV), and it is due to compensation of oxygen deficiencies in the subsurface. The main and lateral oxygen peaks of molybdenum oxide are usually found at 530.5 eV and 532 eV [6,7], respectively, which are in good agreement with our case. The deconvolution process of the O 1s spectrum reveals a further peak at 533.1 eV, which might indicate the existence of ionization associated with weakly adsorbed species. [7] However, the presence of this component can also be ascribed to organic contamination or adsorbed water molecules. [5,8] Moreover, a weaker component was disclosed at lower energies (529.0 eV), and in [5] it has been associated to defective Mo oxides.

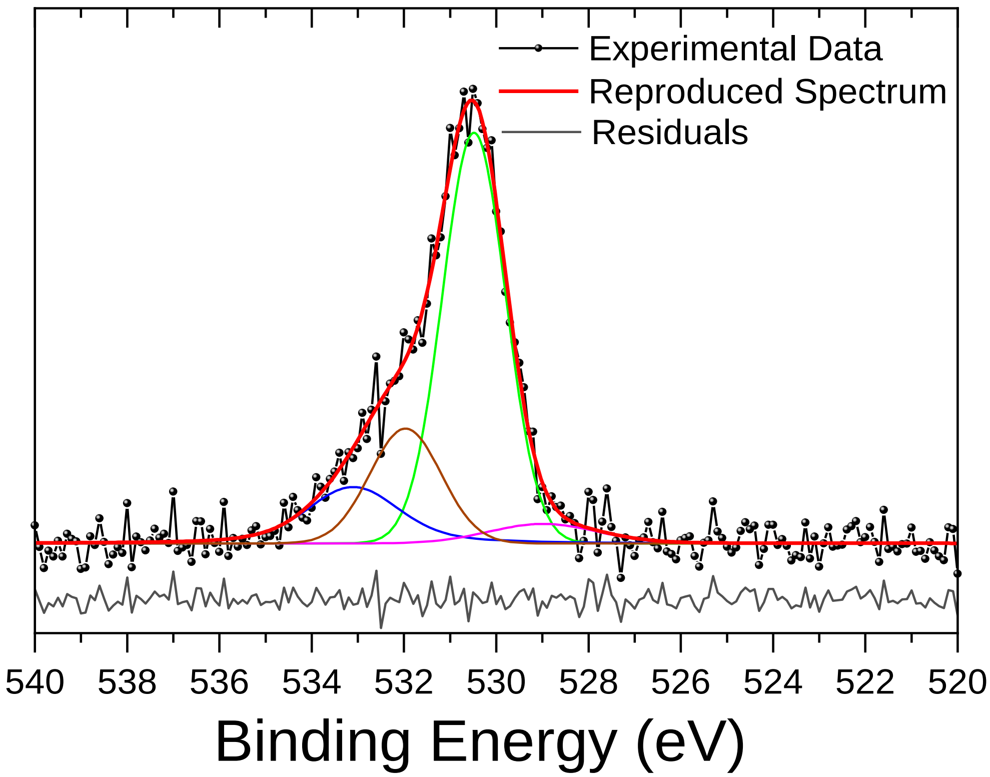

Figure S10: XPS spectra of MoO_x buffer layer in the energy range of O 1s specie.

Peak Type	Area Fit	Center Grvty	Max Height	FWHM
Voigt	519.23814	530.48269	302.28383	1.61357
Voigt	122.45334	533.10096	41.51158	2.36898
Gaussian	42.11851	528.98293	14.38186	2.75122
Gaussian	163.64768	531.96711	84.49699	1.81943

Table S6: Spectral fitting parameters of the XPS spectra in Figure S10.

	Peak Type	Area Fit	Center Max	FWHM
O 1s	Voigt	441.0	530.1	1.9
Peak 1	Voigt	225.1	531.6	2.4
Peak 2	Voigt	156.8	532.5	2.8
V (V) $2p_{1/2}$	Voigt	60.0	524.5	2.1
V (III) $2p_{1/2}$	Voigt	16.8	523.1	1.6
V (V) $2p_{3/2}$	Voigt	126.4	516.9	1.9
V (III) $2p_{3/2}$	Voigt	42.1	515.4	1.5

Table S7: Spectral fitting parameters related to the fitting process of the V_2O_x film.

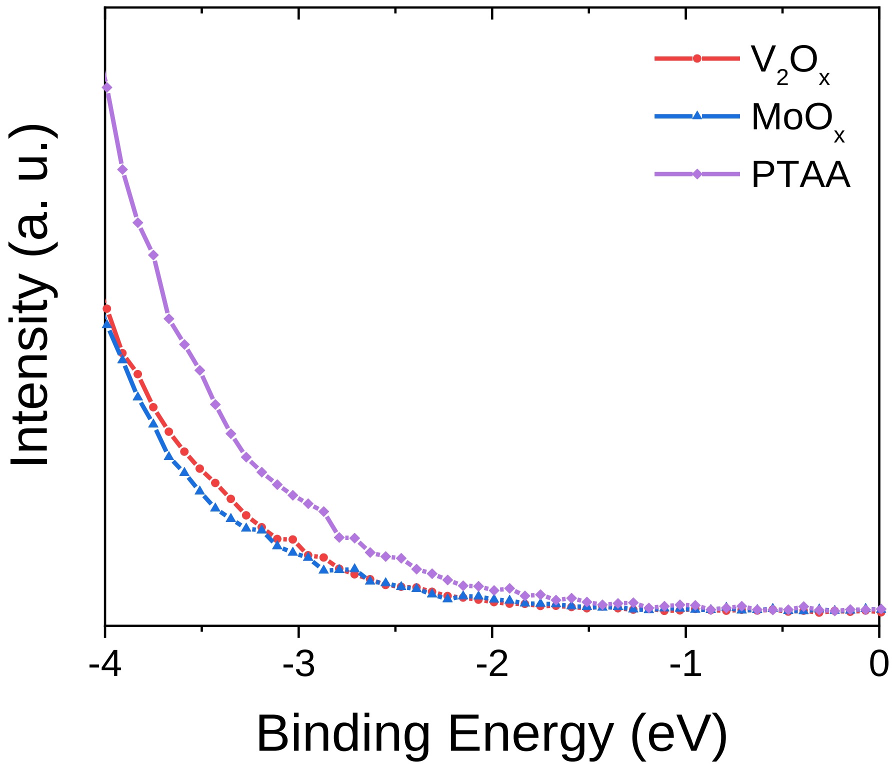

Figure S11: UPS spectra of PTAA, MoO_x, V_2O_x.

From this spectra, the calculated work function values are:

SAMPLE	INTERSECTION (eV)	WORK FUNCTION (eV)
gl/ITO/PTAA	1.2	5.25
gl/FTO/ETL/PVK/PTAA/MoO_x	1.25	5.3
gll/FTO/ETL/PVK/PTAA/V_2O_x	1.35	5.4

Table S8: Work function values for the PTAA, MoO_x and V_2O_x layers. The intersection values are also reported.

A schematization of the obtained energy alignment is also reported:

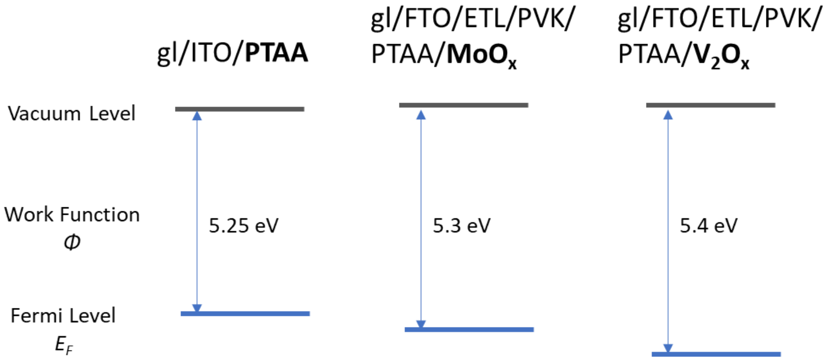

	V_{OC} (V)	J_{SC} (mA/cm^2)	FF (%)	PCE (%)
ST-PSCs without PBL	0.85 ± 0.12	21.97 ± 1.02	44.90 ± 3.50	8.51 ± 1.84
ref. Au (batch V_2O_x)	1.00 ± 0.03	22.70 ± 0.53	75.83 ± 2.01	16.75 ± 0.72
V_2O_x – 2.5 nm	0.97 ± 0.01	22.11 ± 0.83	68.91 ± 3.55	14.73 ± 0.89
V_2O_x – 5 nm	1.01 ± 0.01	20.64 ± 0.68	64.50 ± 3.05	13.41 ± 0.86
V_2O_x – 7.5 nm	0.93 ± 0.03	20.65 ±0.86	59.85 ± 6.67	11.59 ± 1.60
V_2O_x – 10 nm	0.82 ± 0.03	10.57 ± 3.51	41.38 ± 8.19	3.78 ± 2.12
ref. Au (batch MoO_x)	1.02 ± 0.01	22.21 ± 0.61	75.60 ± 3.49	17.17 ± 0.75
MoO_x – 2.5 nm	0.98 ± 0.02	20.52 ± 0.30	59.45 ± 4.32	12.02 ± 1.18
MoO_x – 5 nm	1.02 ± 0.02	20.62 ± 0.34	69.80 ± 5.69	14.64 ± 1.12
MoO_x – 7.5 nm	1.00 ± 0.02	20.99 ± 0.40	74.23 ± 1.54	15.70 ± 0.53
MoO_x – 10 nm	1.008 ± 0.004	21.31 ± 0.16	67.30 ± 2.52	14.46 ± 0.55

Table S9: Mean and standard deviation of the electrical parameters of the ST-PSCs without PBL and with different thicknesses of PBLs. The parameters of the gold-based reference for the batch on V_2O_x experiment and the batch on MoO_x experiment are reported. The experiments were conducted on 10 samples for each category.

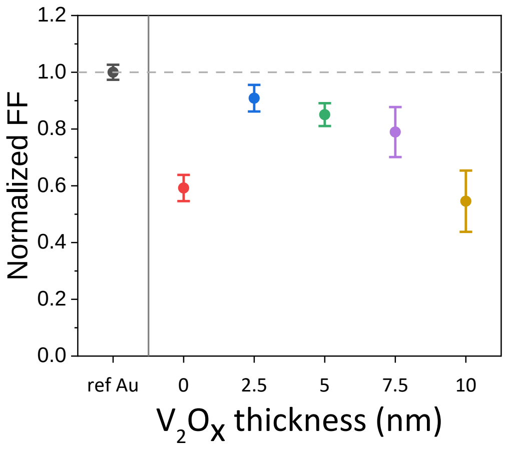

Normalized FF
ref Au
0
2.5
5
7.5
10
V2Ox thickness (nm)

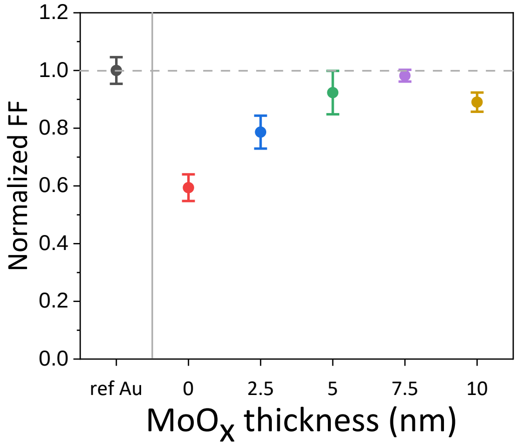

Normalized FF
ref Au
0
2.5
5
7.5
10
MoOx thickness (nm)

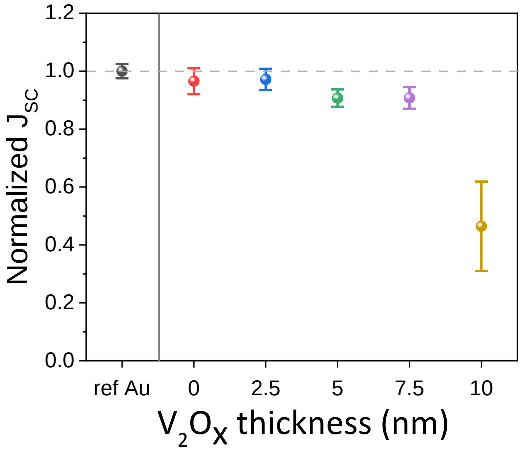

Normalized JSC
ref Au
0
2.5
5
7.5
10
V2Ox thickness (nm)

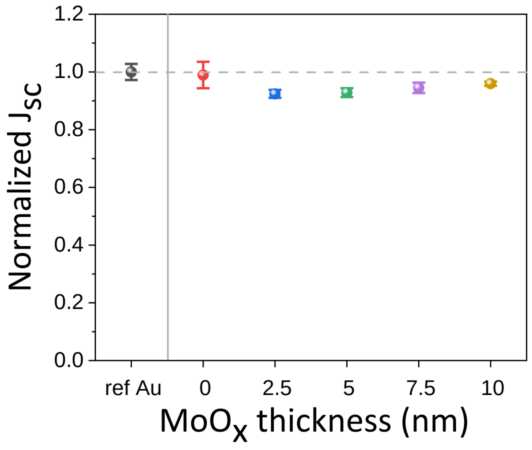

1.2
1.0
0.8
0.6
0.4
0.2
0.0
Normalized J_{SC}
ref Au
0
2.5
5
7.5
10
MoO_X thickness (nm)

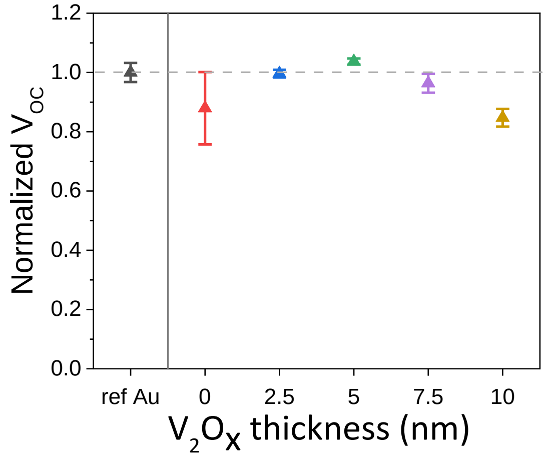

1.2
1.0
0.8
0.6
0.4
0.2
0.0
Normalized V_{OC}
ref Au
0
2.5
5
7.5
10
V_2O_X thickness (nm)

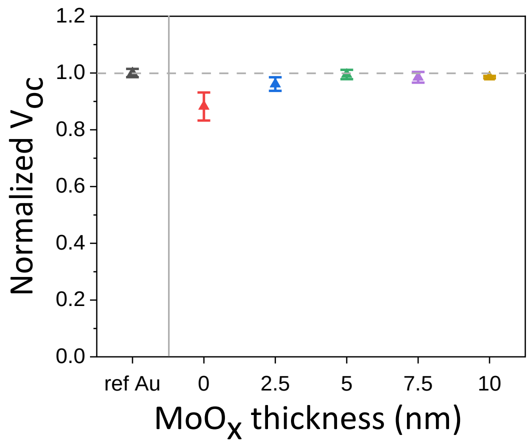

1.2
1.0
0.8
0.6
0.4
0.2
0.0
Normalized V_{OC}
ref Au
0
2.5
5
7.5
10
MoO_X thickness (nm)

Figure S12: Normalized fill factor, short circuit current and open circuit voltage as a function of the PBL thickness for V_2O_x- and MoO_x-based samples. The opaque devices, topped with gold (Au), are the reference samples.

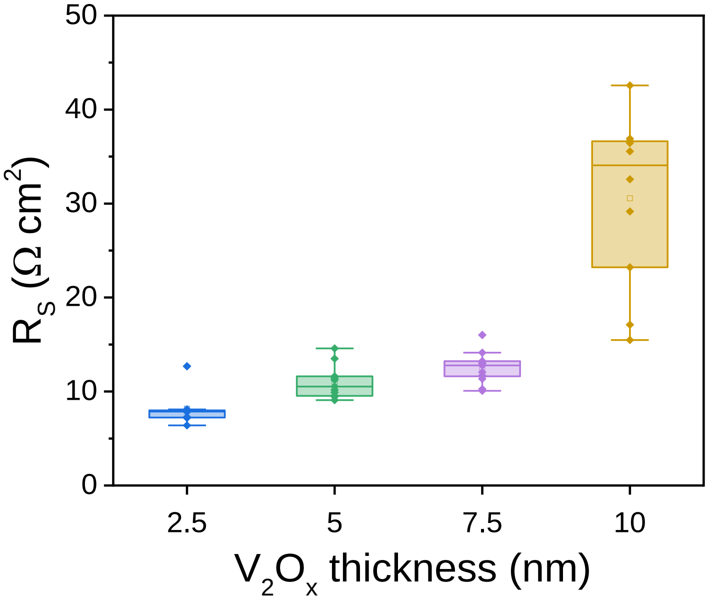

Figure S13: Series resistance (R_S) box plot as a function of the V_2O_x thickness, employed as PBL in semi-transparent perovskite solar cells under test.

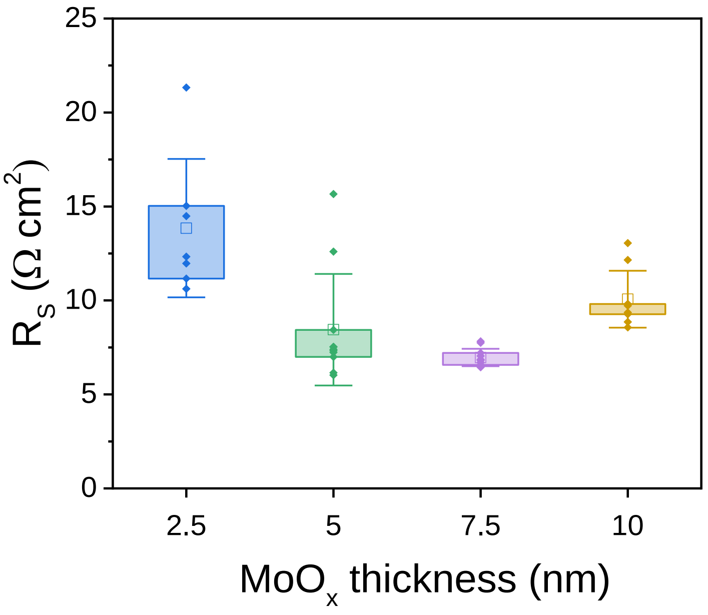

Figure S14: Series resistance (R_S) box plot as a function of the MoO_x thickness, employed as PBL in semi-transparent perovskite solar cells under test.

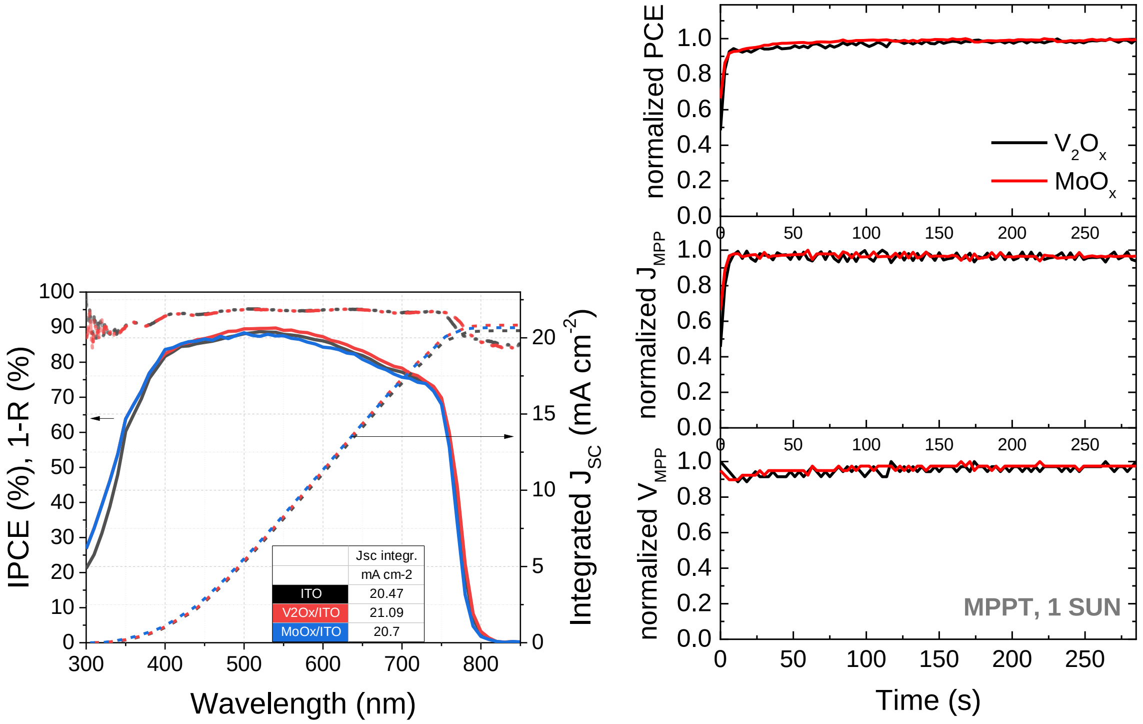

Figure S15: On the left side, EQE, 1-R and integrated short circuit current as a function of wavelength of devices without PBL (ITO) and with PBL (V_2O_x/ITO, MoO_x/ITO). On the right side, maximum power point tracking over time of samples with the PBLs.

	**n**	**JO pA/cm2**	**RS Ω cm2**	**RP kΩ cm2**								
**With PBL**	1.5	0.1	4	15.3								
	nA	JO_A pA/cm2	RS Ω cm2	RP_A Ω cm2	RS_A Ω cm2	JO_B nA/cm2	RS_B Ω cm2	nC	JO_C pA/cm2	RS_C Ω cm2	RP_B kΩ cm2	RP_C kΩ cm2
**Without PBL**	1.6	0.1	5.85	270	0.45	11	9	2.2	11	0.9	1	7

Table S10: Summarized electrical parameters extrapolated from the simulations with LTSpice.

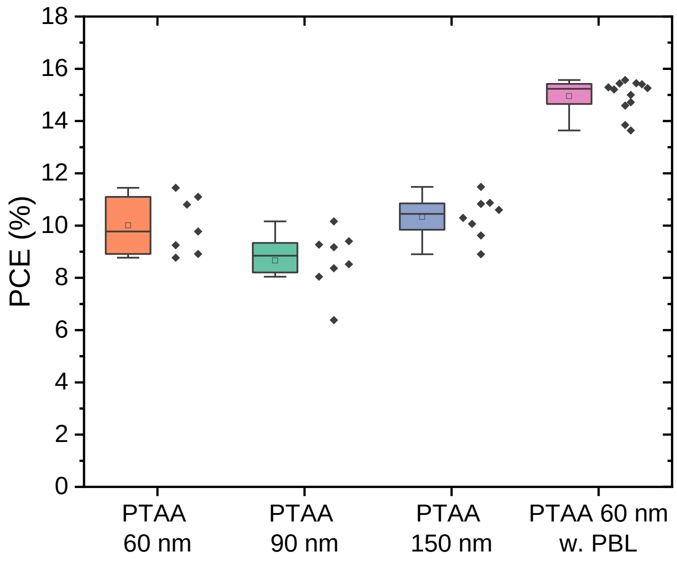

Figure S16: PCEs of ST-PSCs with different PTAA thickness (60 nm, 90 nm, 150 nm) without PBL and 60 nm thick-PTAA with PBL deposited on top prior to ITO sputtering.

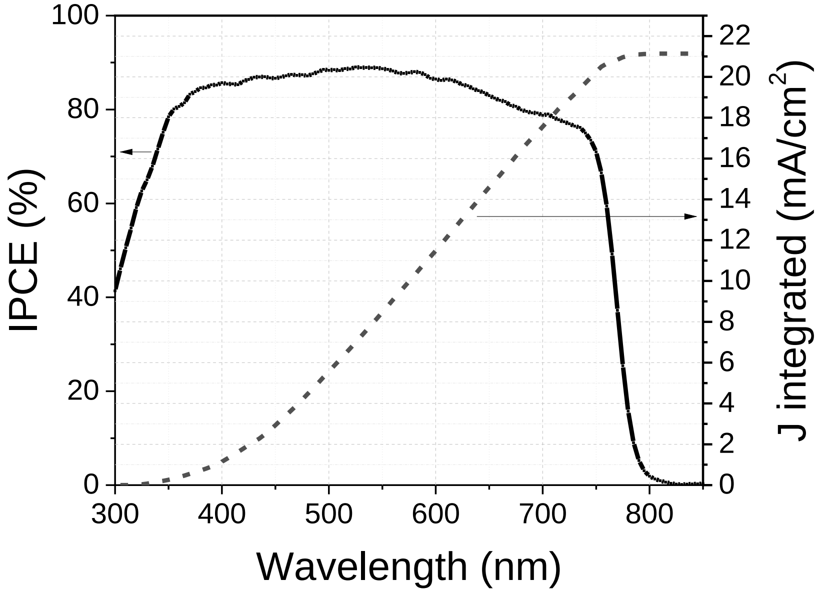

Figure S17: IPCE of the large area ST-PSC.

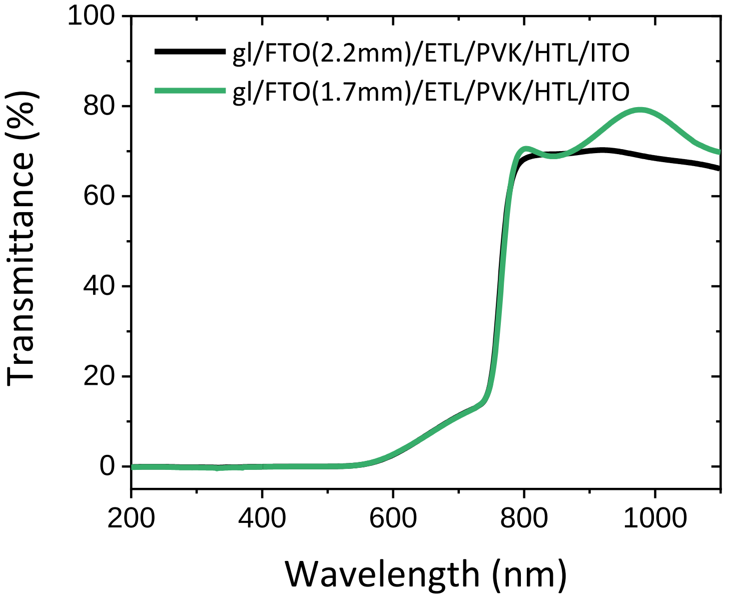

Figure S18: Transmittance of a complete semi-transparent device on 2.2 mm-thick (black curve) and 1.7 mm-thick (green curve) FTO-coated glass substrates.

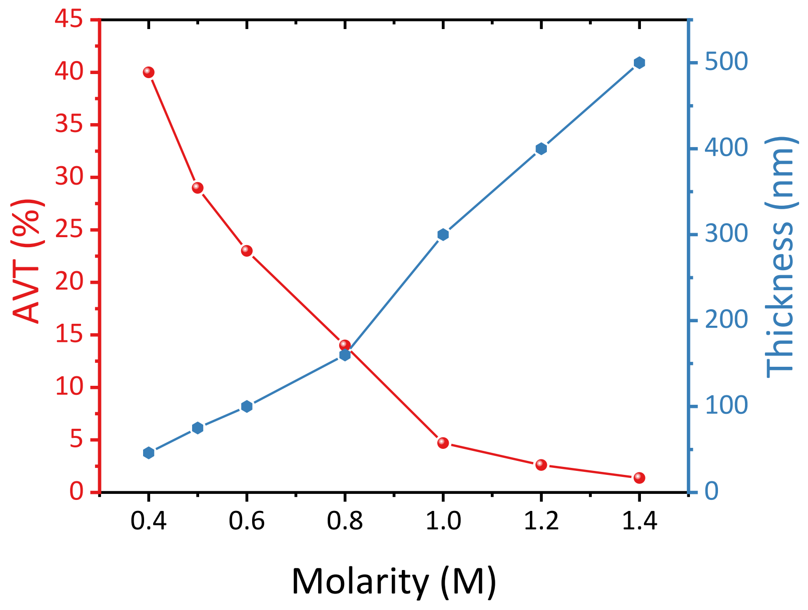

Figure S19: Average Visible Transmittance (AVT, on the left) and perovskite thickness (on the right) as a function of the concentration of the perovskite solution of the ST-PSCs.

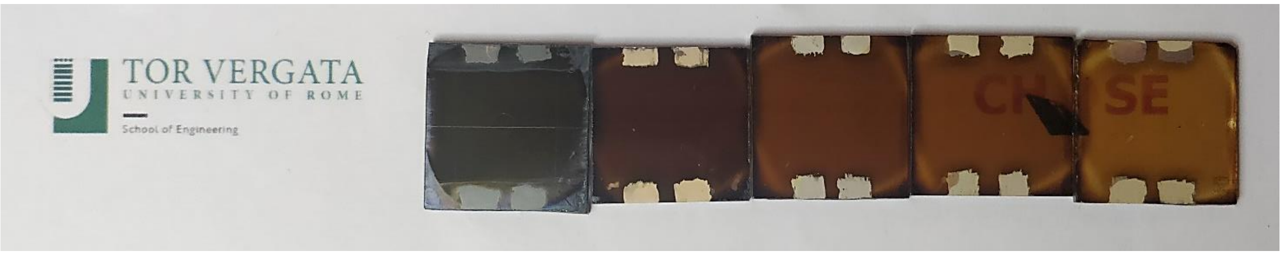

Figure S20: ST-PSCs with different perovskite thicknesses (500 nm, 160 nm, 100 nm, 75 nm, 50 nm) prior to ITO sputtering deposition.

Perovskite thickness	PBL	V_{OC} (V)	J_{SC} (mA cm^{-2})	FF (%)	PCE (%)	AVT (%)
**160 nm**	w/o	0.88	15.8	51.25	7.16	11.37

	w	1.06	15.5	60.56	9.98	10.63
**100 nm**	w/o	0.87	12.42	50.50	5.46	21.48
	w	1.03	12.80	60.84	8.05	20.70
**75 nm**	w/o	0.83	11.31	50.99	4.78	30.08
	w	1.02	10.81	60.5	6.73	30.87
**50 nm**	w/o	0.69	9.07	50.17	3.14	42.10
	w	0.82	9.38	48.7	3.76	42.20

Table S11: Electrical parameters extrapolated from the J-V curves of Fig. 8-B in the main text. The AVT of the ST-PSCs is also reported.

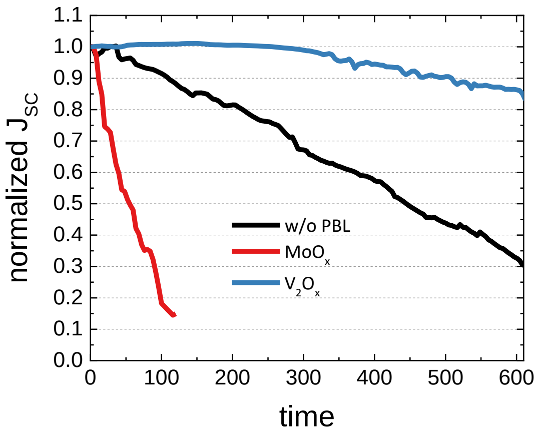

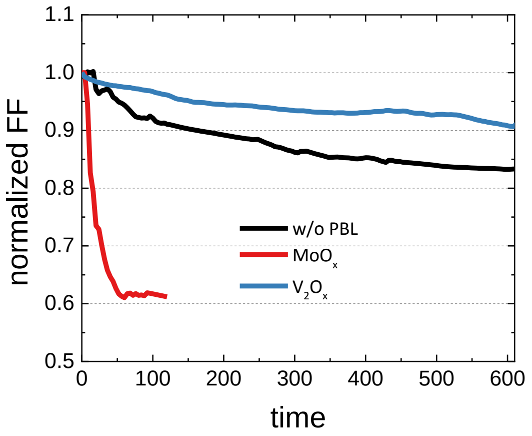

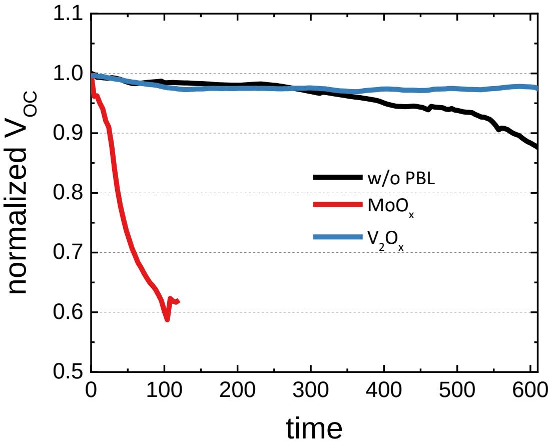

Figure S21: Normalized J_{SC}, FF and V_{OC} of encapsulated ST-PSCs with and without PBL under light soaking.

REFERENCES

1. Meyer, J., Hamwi, S., Kröger, M., Kowalsky, W., Riedl, T., and Kahn, A. (2012) Transition metal oxides for organic electronics: Energetics, device physics and applications. *Adv. Mater.*, **24** (40), 5408–5427.

2. Ikeda, T., Baba, M., and Kqshida, N. (1994) Transition-metal oxide resists for electron-beam and focused-ion-beam lithography. *J. Photopolym. Sci. Technol.*, **7** (3), 585–594.

3. Best, S.A., Squires, R.G., and Walton, R.A. (1979) The X-Ray Photoelectron Spectra of Heterogeneous Catalysts. *J. Catal.*, **60**, 171–183.

4. Sun, Y., Hu, X., Luo, W., and Huang, Y. (2012) Ultrafine MoO 2 nanoparticles embedded in a carbon matrix as a high-capacity and long-life anode for lithium-ion batteries. *J. Mater. Chem.*, **22** (2), 425–431.

5. Vernickaitė, E., Lelis, M., Tsyntsaru, N., Pakštas, V., and Cesiulis, H. (2020) XPS studies on the Mo oxide-based coatings electrodeposited from highly saturated acetate bath. *Chemija*, **31** (4), 203–209.

6. Ramana, C. V., Atuchin, V. V., Pokrovsky, L.D., Becker, U., and Julien, C.M. (2007) Structure and chemical properties of molybdenum oxide thin films. *J. Vac. Sci. Technol. A Vacuum, Surfaces, Film.*, **25** (4), 1166–1171.

7. Dupin, J.C., Gonbeau, D., Vinatier, P., and Levasseur, A. (2000) Systematic XPS studies of metal oxides, hydroxides and peroxides. *Phys. Chem. Chem. Phys.*, **2** (6), 1319–1324.

8. Silversmit, G., Depla, D., Poelman, H., Marin, G.B., and De Gryse, R. (2004) Determination of the V2p XPS binding energies for different vanadium oxidation states (V5+ to V0+). *J.*

Electron Spectros. Relat. Phenomena, **135** (2–3), 167–175.
